# *Fam20c* regulates the calpain proteolysis system through phosphorylating Calpasatatin to maintain cell homeostasis

**DOI:** 10.1186/s12967-023-04275-4

**Published:** 2023-06-27

**Authors:** Xinpeng Liu, Lili Jiang, Wenxuan Zhang, Jiahui Zhang, Xinrui Luan, Yuanbo Zhan, Tuo Wang, Junlong Da, Lixue Liu, Shujian Zhang, Yuyao Guo, Kai Zhang, Zhiping Wang, Nan Miao, Xiaohua Xie, Peihong Liu, Ying Li, Han jin, Bin Zhang

**Affiliations:** 1 Heilongjiang Provincial Key Laboratory of Hard Tissue Development and Regeneration, The Second Affiliated Hospital of Harbin Medical University, Harbin, China; 2 Department of Oral and Maxillofacial Surgery, Stomatological Hospital, School of Stomatology, Southern Medical University, Guangzhou, 510280 China; 3 Department of Pediatric Dentistry, School of Stomatology, The First Affiliated Hospital of Harbin Medical University, Harbin, China; 4 Department of Stomatology and Dental Hygiene, The Fourth Affiliated Hospital, Harbin Medical University, Harbin, China; 5 Department of Periodontology and Oral Mucosa, The Second Affiliated Hospital of Harbin Medical University, Harbin, China; 6 Department of Implantology, School and Hospital of Stomatology, Cheeloo College of Medicine, Shandong University, Jinan, Shandong 250012 People’s Republic of China; 7 Department of Stomatology, The Second Affiliated Hospital of Harbin Medical University, Harbin, China; 8 Department of Stomatology, The First Affiliated Hospital of Harbin Medical University, Harbin, China; 9 Heilongjiang Academy of Medical Sciences, Harbin, China

## Abstract

**Background:**

The family with sequence similarity 20-member C (FAM20C) kinase, a Golgi casein kinase, which is responsible for phosphorylating the majority of the extracellular phosphoproteins within S-x-E/pS motifs, and is fundamentally associated with multiple biological processes to maintain cell proliferation, biomineralization, migration, adhesion, and phosphate homeostasis. In dissecting how FAM20C regulates downstream molecules and potential mechanisms, however, there are multiple target molecules of FAM20C, particularly many phenomena remain elusive, such as changes in cell-autonomous behaviors, incompatibility in genotypes and phenotypes, and others.

**Methods:**

Here, assay for transposase-accessible chromatin using sequencing (ATAC-seq), RNA sequencing (RNA-seq), proteomics, and phosphoproteomics were performed in *Fam20c*-dificient osteoblasts and to facilitate an integrated analysis and determine the impact of chromatin accessibility, genomic expression, protein alterations, signaling pathway, and post translational modifcations.

**Results:**

By combining ATAC-seq and RNA-seq, we identified TCF4 and Wnt signaling pathway as the key regulators in *Fam20c*-dificient cells. Further, we showed Calpastatin/Calpain proteolysis system as a novel target axis for FAM20C to regulate cell migration and F-actin cytoskeleton by integrated analysis of proteomics and phosphoproteomics. Furthermore, Calpastatin/Calpain proteolysis system could negatively regulate the Wnt signaling pathway.

**Conclusion:**

These observations implied that *Fam20c* knockout osteoblasts would cause cell homeostatic imbalance, involving changes in multiple signaling pathways in the conduction system.

**Supplementary Information:**

The online version contains supplementary material available at 10.1186/s12967-023-04275-4.

## Introduction

The family with sequence similarity 20C (FAM20C), the *bona fide* “Golgi casein kinase”, answers for the phosphorylation of over 100 secreted phosphoproteins with a consensus motif Ser-x-Glu/pSer and is ubiquitously expressed in multiple tissues and bodily fluids [1–4]. Moreover, FAM20C figures prominently in a wide range of cellular processes, including phosphate metabolism, lipid homeostasis, wound healing, biomineralization, cardiac function, cell adhesion, and migration [1, 5–8]. These abundant and useful functions make FAM20C an important protein kinase. At the physiological and pathological level, FAM20C gene mutations or aberrant function of kinase lead to many diseases, such as Rains Syndrome, cancers, and other diseases. Notwithstanding, the exploration of the pivotal role of FAM20C in health and disease remains in an infant stage.

Aberrant FAM20C mutation causes lethal or non-lethal Raine syndrome in humans, which is clinically characterized by generalized osteosclerotic dysplasia and hypophosphatemia [9, 10]. To better understand the pathogenesis of Raine syndrome in humans, from work in animal and cellular models established by ablating *Fam20c* gene, diseases and phenotypes associated with Raine syndrome have been studied. Mice lacking *Fam20c* present the phenotype similar to human non-lethal Raine syndrome, including growth retardation, rickets/osteomalacia, defects in the growth plate, defective osteocytes differentiation, reduction of serum phosphate, and elevation of serum fibroblast growth factor (FGF23) [11]. This has been partially attributed to elevated FGF23, which regulates serum phosphate levels, and its activity is regulated by FAM20C-mediated phosphorylation at Ser180 and O-glycosylation [6, 12]. Furthermore, *Fam20c*^−/−^ mice develop abnormal dental morphological changes and differentiation defects, which can be caused by the suppressing of BMP signaling pathways [13–15]. Thus, considering *Fam20c*-deficient mice develop hard tissue dysplasia and FAM20C is highly expressed in bone and tooth tissues [2, 16, 17], studies have sought to identify the cellular changes in FAM20C ablation cells. Intriguingly, in *Fam20c*-deficient dental mesenchymal cells and osteoblasts, changes in gene expression of key factors related to osteogenesis and mineralization are consistent with *Fam20c*^−/−^ mice, and impaired cellular mineralization could not be rescued by the addition of normal bone-derived extracellular matrix proteins [18, 19]. These findings suggest that FAM20C regulates osteoblast behaviors in a cell-autonomous manner. However, little is known regarding the mechanisms by which FAM20C influences cell autonomous behaviors.

A comprehensive understanding of human diseases requires exploration of their complexity and heterogeneity of the pathogenesis at the epigenetic, genomic, and proteomic levels [20, 21]. In the study of Raine syndrome regard, only 70 cases have been reported so far due to its extremely rare and low incidence rate [22], making it difficult to apply high-throughput sequencing technology in the research field of Raine syndrome. To investigate the mechanisms underlying the FAM20C-associated phenotype in Raine syndrome, some studies have been conducted to study the biological functions of FAM20C by using high-throughput sequencing technology. Zhang et al. [23] analyzed the proteins that interact with FAM20C in the endoplasmic reticulum (ER) and Golgi apparatus through immunoprecipitation and mass spectrometry, revealing that FAM20C could phosphorylate ER oxidoreductin 1α (Ero1α) to maintain the ER redox homeostasis. Besides, our previous research conducted a transcriptional analysis of *Fam20c*-deficient osteoblasts, indicating the cells undergo mesenchymal to epithelial transformation [24]. Besides a single sequencing method, the combination of multi-omics analysis provides a more comprehensive picture linking “molecular information” to “phenotype” through functional and signaling networks [25–27]. Little is known, however, about multi-omics analysis of FAM20C remains to be determined, especially chromatin sequencing.

Here, we use established *Fam20c*-deficient cells in our previously published work [28] to conduct a comprehensive omics analysis. To probe the relationship between chromatin, transcriptional and protein changes response to *Fam20c* ablation in osteoblasts, we performed integrated omics analyses using an assay for transposase-accessible chromatin using sequencing (ATAC-seq), RNA sequencing (RNA-seq), proteomics, and phosphoproteomics. *Fam20c* ablation specifically regulates a subset of genes comprising Wnt signaling pathway molecules and TCF4 was highly enriched in the chromatin opened of *Fam20c* deficient osteoblasts, as revealed by ATAC-seq. We identified Calpastatin/Calpain proteolysis system as a novel target axis for FAM20C to regulate cell migration and F-actin cytoskeleton. Subsequently, we demonstrated that Calpain agonist treatment rescued *Fam20c*-deficient cells migration in vitro, and negatively manage the Wnt signaling pathway. Further, our results suggested that the homeostatic imbalance of *Fam20c* knockout osteoblasts may involve changes in multiple signaling pathways in the conduction system.

## Materials and methods

### Cell lines and cell culture

Immortalized mouse *Fam20c*^*f/f*^ osteoblast cells (referred as “OB *Fam20c*^*f/f*^”) were purchased from Applied Biological Materials Inc. (Lot# T0038). *Fam20c*-deficient osteoblasts (referred as “OB *Fam20c*^*KO*^”) were established with infection of Cre recombinase lentivirus as previously published work described [28]. OB *Fam20c*^*f/f*^ and OB *Fam20c*^*KO*^ were cultured at 37 °C with 5% CO_2_. All cells were cultured in α-Minimum Essential Medium (α-MEM) (Biosharp, BL306A) containing 10% fetal bovine serum (Gibco, #10091148) and 1% penicillin–streptomycin (Gibco, #15140122). Additionally, cells were passaged with 0.25% trypsin (Gibco, #12604013).

### ATAC-seq

Cell samples for ATAC-seq were prepared as described in *Current protocols in molecular biology* by Buenrostro et al. [29]. In brief, 50,000 cells were obtained from each sample, resuspended in cold PBS, and centrifugated at 500 g for 5 min at 4 °C. Cell pellets were washed once with 50 μl cold PBS on ice, followed by a 5 min centrifugation at 4 °C. Pelleted nuclei were taken for transposition reaction with Tn5 enzyme, and the DNA fragments after transposition were recovered with MinElute PCR Purification Kit (Qiagen). Accessible DNA was amplified by PCR with 1 X NEBNext High-Fidelity Pcr Master Mix (New England Biolabs, MA). Libraries were sequenced on a Novaseq 6000 (Illumina).

ATAC raw reads data from each sample was filtered using Trimmomatic software (Version 0.36) [30], after data filtering, the overall quality of the resulting clean reads was assessed. The clean reads were then aligned to reference genome *mm10_gencode* using the BWA program (Version 0.7.13-r1126). Three repeated samples for each group were used for callpeak by MACS2 (Version 2.1.2) [31] with the parameters qvalue < 0.05. Reads distributions across genes were presented using deeptools (Version 3.4.3)[32], and genes were represented as lines sorted in descending order based on the signal strength in the heatmap. Motif analysis was performed using the HOMER's findMotifsGenome.pl tool (v4.11). Differential accessible peak analysis was performed by using bedtools software [33] and DESeq2 (Version 1.16.0)[34], log2 fold change ≥ 1 or ≤ -1, and p-value < 0.05 were considered as the cutoff values. The raw data and processed data were uploaded into the Gene Expression Omnibus (GEO) database (https://www.ncbi.nlm.nih.gov/geo/) with an accession number GSE233445.

Gene Ontology (GO) and Kyoto gene and genome encyclopedia (KEGG) signal pathway analysis of differential peaks nearest genes were performed using ClusterProfiler with a False Discovery Rate (FDR)-adjusted p-value cutoff of 0.05. Thus the significant GO categories and pathways were identified.

### Integration Analysis of ATAC-seq and RNA-seq

RNA-seq analysis was as previously reported [28]. The associated genes in open chromatin regions with enhanced and attenuated ATAC-seq signal overlapped with the up and down differentially expression genes (DEGs) in the transcriptomes, respectively. Further, GO and KEGG pathways analyses were performed as described above.

### Proteomic and phosphoproteomic analysis

Cells were washed three times with cold PBS and lysed in lysis buffer containing 50 μl 50 mM Tris–HCl (pH 8.5), 57 μl ddH_2_O, 10 μl 400 mM 2-chloroacetamide (CAA) (Sigma, 22,790), 2 μl tris (2-carboxyethyl) phosphine (Sigma, 646547) and 1 μl phosphatase inhibitor cocktail. Samples were then boiled in a 95 °C water bath for 10 min in darkness. Subsequently, cooled at room temperature and diluted five-fold. The protein concentration was measured by using BCA protein assay, and the rest part of protein samples was digested with Lys-C (Wako) at 37 °C for 3 h. Protein samples underwent trypsin digestion (1:50) at 37 °C overnight. Afterward, samples were acidified by 1% trifluoroacetic acid (TFA) and ethyl acetate. The mixture of lysates was centrifuged at 15000*g* for 3 min, the bottom aqueous phase was collected followed by vacuum-dried centrifugation and desalting. 95% and 5% portions of each sample were used for phosphoproteomics and proteomics experiments, respectively.

Peptide samples were analyzed on the nanoElute coupled online with a timsTOF Pro mass spectrometer (Bruker). Peptides were re-dissolved in 0.1% formic acid (FA) and loaded onto a 25 cm in-house column n (360 μm OD × 75 μm inner diameter) packed with C18 resin (particle size 2.2 μm, pore size 100 Å, Michrom Bioresources). All peptides were separated onto the analytical column with a 120 min gradient (buffer A: 0.1% FA in ultrapure water; buffer B: 0.1% FA in acetonitrile) at a constant flow rate of 300 nL/min (90 min, 0 to 37% buffer B using a linear AB gradient of 2 to 22% of buffer B; 10 min, 22 to 37% of buffer B, 10 min, 37 to 80% of buffer B; 10 min, 80% of buffer B). Mass spectrometry was set under a data-dependent acquisition mode. Mass range was 100–1700 m/z at a resolution of 40,000.

MS raw files produced by LC–MS/MS were searched against the Uniprot human proteome database using PEAKS Studio X + software (Bioinformatics Solutions Inc.). Mass tolerances were 15 ppm for initial precursor mass and 6 ppm for final tolerance. Carbamidomethyl of cysteine (+ 57.0214 Da) was considered a fixed modification. Variable modifications were acetylation (+ 42.011 Da) at the N terminus of proteins, oxidation (+ 15.9949 Da) on methionine residues, and phosphorylation (+ 79.996 Da) on serine, threonine, or tyrosine residues. Up to 2 missed cleavages were allowed. The cutoff of FDR was 1% for all proteins, peptides, and phosphosites. Differential expression analyses were determined based on p-value < 0.05 and |log2(Fold change)|> 1.

### Cell migration assays.

To measure the relative migration ability of cells, scratch-wound healing assays were performed. Cells were plated in a 6-well plate at a density of 50*10^4^/well. After cells reached confluency, manually scraped the monolayer cell with a 10 μl pipette tip to create the wounds. The cells were then washed with phosphate saline buffer (PBS), replenished with serum-free α-MEM, photographed with the inverted phase-contrast microscope, and recorded the exact location of each wound. Afterward, the cells were placed back in the cell-culture incubator, and the recorded scratch regions were photographed 24 h after wounding. For statistical analysis, using Image J to outline the pictures, measure the area of the scratch, analysis and plot were performed using GraphPad Prism 8.

### Phalloidin-fluorescent staining

Cells were seeded in a 24-well plate at approximately 100% confluence and subsequently scraped with the pipette tip to create wounds. For scratch-wound healing experiments, cells were treated as described above. At 24 h after wounding, cells were fixed by 4% formaldehyde solution for 10 min, and washed three times with PBS, each time for 5 min. Cells were then permeabilized with 0.1% Triton X-100 for 5 min, and washed three times with PBS. To reduce nonspecific background staining, cells were blocked in 1% bovine serum albumin (BSA) for 30 min at room temperature. The rhodamine phalloidin staining solution (MESGEN, MF8204) was diluted in 1% BSA and incubated cells for 20 min at room temperature. The cells were rinsed by PBS three times, each time for 5 min. 200 μl per well of Hoechst 33,258 (Beyotime, C1018) was used to label the nuclear of cells. Cells were observed on fluorescence microscope (Nikon), and photographed at least three different representative regions at 4× magnification or 10× magnification.

### Quantitative real-time PCR analysis

RNA was extracted from cells by using RNAiso Plus reagent (Takara, 9108) following the manufacturer’s protocols. RNA quality and concentration were determined by using a Nanovue spectrophotometer (GE Healthcare Life Sciences, Marlborough, MA, USA). cDNA was prepared by RNA reverse transcripted with PrimeScript RT reagent kit (Takara, RR047A). Subsequently, SYBR TB Green Premix Ex Taq™ kit (Takara, RR820A) was used for one-step real-time RT-PCR analysis on the MxPro-Mx3000P real-time PCR System. The experiment was repeated three times.

Normalize the expression value of the target gene in a given sample to the corresponding GAPDH expression. The relative expression value of the targeted genes was calculated by the 2 ^− △ △^Ct method. The primers were: *RhoA*-F, 5’- GAAACTGGTGATTGTTGGTGATG-3’, *RhoA*-R, 5’- ACCGTGGGCACATAGACCT-3’, *Rac1*-F, 5’- ACGGAGCTGTTGGTAAAACCT-3’, *Rac1*-R, 5’- AGACGGTGGGGATGTACTCTC-3’, *Cdc42*-F, 5’- CCCATCGGAATATGTACCAACTG-3’, *Cdc42*-R, 5’- CCAAGAGTGTATGGCTCTCCAC-3’, *Tcf4*-F, 5’-GATGGGACTCCCTATGACCAC-3’, *Tcf4*-R, 5’- GAAAGGGTTCCTGGATTGCCC-3’, *β-catenin*-F, 5’- ATGGAGCCGGACAGAAAAGC-3’, *β-catenin*-R, 5’- TGGGAGGTGTCAACATCTTCTT-3’, *Calpain1*-F, 5’- ATGACAGAGGAGTTAATCACCCC-3’, *Calpain1*-R, 5’- GCCCGAAGCGTTTCATAATCC-3’, *Calpain2*-F, 5’- GGTCGCATGAGAGAGCCATC-3’, *Calpain2*-R, 5’- ATGCCCCGAGTTTTGCTGG-3’, *Calpastatin*-F, 5’- GGAAGGACAAACCAGAGAAGC-3’, *Calpastatin* -R, 5’- AGGGGCAGCTATCCAAATCTT-3’, *GSK-3β*-F, 5’- TTGGACAAAGGTCTTCCGGC-3’, *GSK-3β*-R, 5’- AAGAGTGCAGGTGTGTCTCG-3’, *GAPDH*-F, 5’- AACTCCCACTCTTCCACCTTC-3’, *GAPDH*-R, 5’- CCTGTTGCTGTAGCCGTATTC-3’.

### Western blot and antibodies

Cells were collected and lysed in radio immunoprecipitation assay (RIPA) lysis buffer (Beyotime, P0013B) containing phenylmethanesulfonyl fluoride (PMSF) (Beyotime, ST506) and phosphatase inhibitor cocktail (Sigma, P5726). Total proteins were extracted in the supernatant. To prepare the nuclear and cytoplasmic extracts, the nuclear and cytoplasmic protein extraction kit (Beyotime, P0028) was applied to lyse the cells following the manufacturer’s instructions. After vigorous vortexing, the lysates were centrifuged at 12000*g* for 5 min at 4 °C. The supernatant was collected as the cytoplasmic protein. Add the nuclear protein extraction reagent to the pellet, vortexing and ice bathing alternately for 30 min, the lysates were centrifuged at 12000*g* for 10 min at 4 °C, and the supernatant was collected as the nuclear fraction. All extracted proteins were quantified using the Enhanced BCA Protein Assay Kit (Beyotime, P0010). The protein concentration between different groups was adjusted to be consistent before boiling.

For western blot assay, 40 µg of protein lysates were separated by sodium dodecyl sulfate–polyacrylamide gel electrophoresis (SDS-PAGE) (Beyotime, P0012AC) and transferred to polyvinylidene difluoride (PVDF) membranes (Biosharp, BS-PVDF-45/BS-PVDF-22). After blocking in 5% non-fat milk (Biosharp, BS102), PVDF membranes were then incubated overnight at 4 °C with primary antibodies and probed for Calpastatin (Cell Signaling Technology, #4146), Calpain 1 (Proteintech, 10538-1-AP), Calpain 2 (Proteintech, 11472-1-AP), β-catenin (Proteintech, 51067-2-AP), Tcf4 (Proteintech, 22337-1-AP), GSK3-β (Proteintech, 22104-1-AP), Lamin B (Wanleibio, WL01775), Rho A (Wanleibio, WL02853), Cdc42 (Proteintech, 10155-1-AP), β-Tubulin (Abmart, M20005), and GAPDH (Proteintech, 10494-1-AP). Peroxidase-conjugated IgG (Biosharp, BL003A) was used as the secondary antibody, and blots were developed using the enhanced chemiluminescence kit (Meilunbio, MA0186) detection.

### Immunofluorescence analysis

Cells were seeded in a 24-well plate at approximately 60% confluence. Cells were fixed by 4% paraformaldehyde (PFA) for 30 min at room temperature, and washed three times with PBS, each time for 5 min. Cells were then blocked in immunol staining blocking buffer (Beyotime, P0102) for 1 h at room temperature. The primary antibodies were diluted in primary antibody dilution buffer for Immunol staining (Beyotime, P0262) and incubated the cells for overnight at 4 °C. PBS rinsed the cells five times, each time for 5 min. Secondary antibodies (Cell Signaling Technology, #8890S) were diluted in antibody dilution buffer (1:400) and incubated for 2 h at room temperature. Hoechst 33258 was used to tag the nuclear of cells. Cells were observed on fluorescence microscope, and photographed at least three different representative regions at 4× magnification or 10× magnification.

### Casein zymogram

To determine calpain activity, casein zymography analysis was performed. The main principle is that the enzymes in the lysates are separated by the polyacrylamide gel containing casein. Casein molecules in zymography gels act as substrates for calpain in solutions containing calcium ions, and their degradation reflects enzymatic activity [35, 36]. In resolving gel, 0.21 mg casein was copolymerized with 4.9 ml ddH_2_O, 2.5 ml 1.5 mol/L Tris–HCl (pH 8.8), 2.5 ml polyacrylamide solution (4:0.16), 40 μl ammonium persulfate solution (APS), and 28 μl N,N,N’,N’-Tetramethylethylenediamine (TEMED). For stacking gel, it contained 6.5 ml ddH_2_O, 2.5 ml 1.32 mol/L Tris–HCl (pH 6.8), 1 ml polyacrylamide solution, 50 μl APS, and 10 μl TEMED. The casein gel was pre-run at 160 V for 30 min at 4 °C with electrode running buffer. The running buffer (10x) contained 25 mM Tris–HCl (pH 8.3), 192 mM glycine, 1 mM ethylene glycol-bis (β-aminoethyl ether)-N’,N’,N’,N’-tetraacetic acid (EGTA), 0.05% (v/v) 2-mercaptoethanol (2-MCE), and 1 mM ethylenediaminetetraacetic acid (EDTA). Subsequently, lysates were loaded with sample buffer (1:3, v/v), which included 150 mM Tris–HCl (pH 6.8), 0.04% (w/v) bromophenol blue, 20% (v/v) glycerol, and 0.75% (v/v) 2-MCE. The gel with samples loaded was run for electrophoresis at 125 V for 3 h at 4 °C. At the end of the electrophoresis, the gel was incubated in proteolysis buffer for 24 h at 20 °C. The proteolysis buffer (10x) was comprised of 20 mM Tris–HCl (pH 7.4), 10 mM dithiothreitol (DTT), and 4 mM CaCl_2_. The gel was stained with Coomassie Blue Fast Staining Solution (Beyotime, P0017) for 60 min, and the thermal cycler imaging system was applied to photograph the bands of the gel.

### Mouse models

All mouse models are on a C57BL6/J background and breading in a SPF grade facility. The generation of conditional knockout mice was as follows: (1) *Fam20c*^*flox/flox*^ mice (Texas A&M University College of Dentistry, America) mated with *Osx-Cre* mice (Biocytogen, China) from the progeny of which the *Fam20c*^*flox/*+^; *Osx-Cre* mice were selected. (2) By mating these mice with *Fam20c*^*flox/flox*^ mice, the *Osx-Cre*; *Fam20c*^*flox/flox*^ mice were obtained from the progeny, whose gene *Fam20c* was deleted in the pre-osteoblast. In this research, *Osx-Cre; Fam20c*^*flox/flox*^ (conditional knockout, cKO) mice were used as the experimental group, and their littermates with *Fam20c*^*flox/flox*^ genotype were put into the control group. The genotype identification was performed on the tails of postnatal mice by polymerase chain reaction, the genotyping of each mouse was repeated three times.

### Immunohistochemical staining

The bone tissue samples of 4-week-old mice were collected and fixed for 48 h, then placed in the decalcified fluid (15% EDTA). After the end of decalcification, the mouse tissues were placed in the automatic tissue dehydrator. The wax-soaked tissues were placed into the embedding machine. Conventional sections of tissue wax blocks were cut with a thickness of 3 µm, and the position and depth of the tissue sections in the cKO group and the control group were basically the same as far as possible. Immunohistochemical staining was performed on 3 µm thickness paraffin sections, the sections were sequentially deparaffinized, washed with pure water, and soaked in 3% H_2_O_2_ to block endogenous peroxidases. Antigen retrieval was performed using citrate buffer under high pressure, and then the slides were incubated in 10% normal goat serum and 5% bovine serum albumin (Solarbio, China) for 60 min, and incubated with the antibody for 2 h at room temperature. The sections were washed in PBST (PBS plus 0.1% Tween) and incubated with the secondary antibody at room temperature for 60 min. Then the slides were washed in PBST, and immunopositive reactions were visualized using a 3,3’-diaminobenzidine tetrahydrochloride solution. The nuclei were counterstained with hematoxylin. The slides were washed in distilled water and dehydrated in graded alcohol, cleared in xylene. Images were taken with a biological microscope (Nikon, Japan).

### Statistical analysis

Each experiment was repeated at least three times. Statistical analysis was performed with GraphPad Prism8.0 software. Student’s t test was used for univariate comparison between two groups. *P* < 0.05 was considered to be different and statistically significant and presented as * *P* < 0.05, ** P < 0.01, or *** *P* < 0.001.

## Results

### Mapping the ATAC-seq landscape of *Fam20c*-deficient osteoblasts

We first sought to map the chromatin landscape underlying the observed FAM20C-related transcriptional programs in the osteoblasts, and thus ATAC-seq was employed. This method for capturing chromatin accessibility regions by Tn5 transposases, which will integrate adapters payload into accessible chromatin regions such as enhancers, promoters, and insulators [37]. We isolated nuclei from OB *Fam20c*^*f/f*^ and OB *Fam20c*^*KO*^ cells. Samples were massively sequenced in parallel, and the high-confidence regions of chromatin accessible regions were identified via overlapping peaks of the replicate samples. For data quality control, we examined whether our data between replicates were of good sequencing quality (Additional file 8: Table S1) and alignment of reads on the reference genome sequence (Additional file 9: Table S2). We then evaluated the length distribution of insert size, the chromatin was fragmented into nucleosome free, mono nucleosome, and di-nucleosome patterns (Additional file 1: Figure S1A). This analysis indicated that all samples present similar distribution of fragment sizes and there was a similar degree for Tn5 transposase access to chromatin, also revealing all sample reads were mainly enriched near the transcriptional start site (TSS) (Additional file 1: Figure S1B, C). Furthermore, examination of the Spearman correlation between samples based on the signals on merged ATAC-seq peaks, showed that the three samples were very close to each other in each group (Additional file 1: Figure S1D, E).

**Fig. 1:**
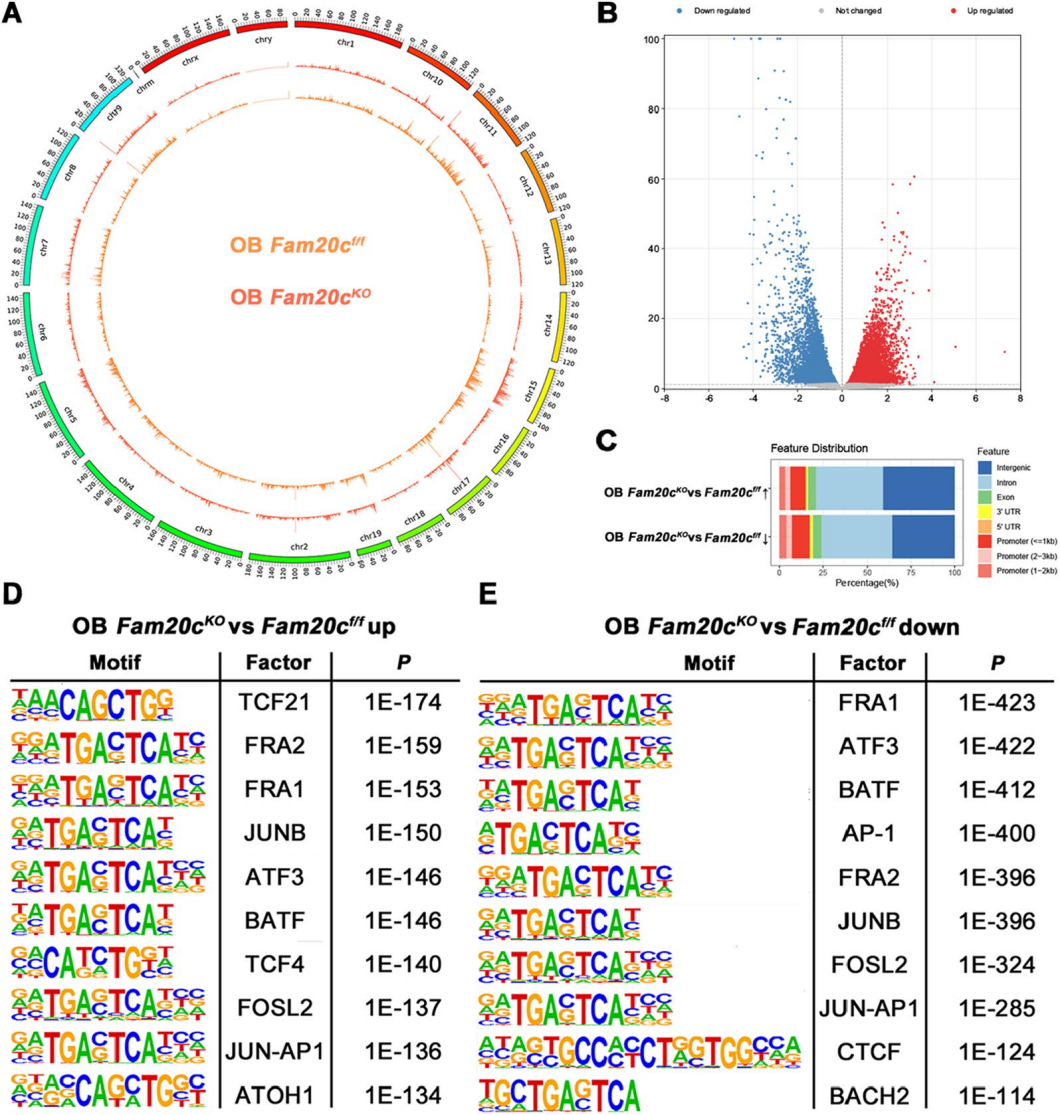
Chromatin accessibility landscapes of *Fam20c*-deficient osteoblasts. **A** Landscape of chromatin accessibility from ATAC-seq analysis in OB *Fam20c*^*f/f*^ and OB *Fam20c*^*KO*^. **B** Volcano plot of all peaks. Differentially accessible regions up-regulated in OB *Fam20c*^*KO*^ compared with OB *Fam20c*^*f/f*^ are indicated as red dots, the differentially accessible regions down-regulated are indicated as blue dots, and the grey dots indicate peaks with no significant difference. The x-axis represents log2 (Fold Change), and the y-axis indicates -log10 (*P* value). **C** Bar chart showing the distribution of up- and down-regulated differentially accessible regions in OB *Fam20c*^*KO*^ relative to gene features. The X-axis represents the proportion of peaks in each functional area, and the Y-axis represents different groups. The genome-wide functional regions were divided into promoter, 5’UTR, 3’UTR, exon, intron, and intergenic regions. **D** Up regulated motifs enriched in OB *Fam20c*^*KO*^ enhancers compared with OB *Fam20c*^*f/f*^ (top 10 motifs displayed). **E** Down regulated motifs enriched in OB *Fam20c*^*KO*^ enhancers compared with OB *Fam20c*^*f/f*^ (top 10 motifs displayed)

Enriched analysis of 9,028 filtered differential accessible peaks created a detailed global overview map between OB *Fam20c*^*f/f*^ and OB *Fam20c*^*KO*^ (Fig. 1A). These peaks were screened based on log2 (Fold Change) > 1 or < -1 and *P* value < 0.05, allowing identification the differentially accessible regions (DARs) between the two groups. Specifically, as shown in Fig. 1B, 5527 (62%) DARs were significantly intensified, and 3501 (38%) DARs were attenuated in *Fam20c*-deficient osteoblasts (Fig. 1B). Having demonstrated that peak annotation could link chromatin accessibility to gene regulation, and classify genome-wide functional regions into promoter, 5’ untranslated region (UTR), 3’ UTR, exon, intron and intergenic [38]. We thus proceeded to test the distribution of DARs on the above genomic features. The results indicated that the genomic distribution of differentially peaks in *Fam20c*-deficient osteoblasts showed a slight shift toward intergenic region and a slight decrease in promoter compared with OB *Fam20c*^*f/f*^ (Fig. 1C). Sequence motif analysis using HOMER revealed a prominent enrichment in OB *Fam20c*^*KO*^ for the basic helix-loop-helix (bHLH) transcription factor family, including transcription 21 (TCF21) and atonal bHLH transcription factor 1 (ATOH1), and the basic leucine zipper (bZIP) transcription factor family including fos-like antigen 2 (FRA2), FRA1, junB proto-oncogene (JUNB), activating transcription factor 3 (ATF3), basic leucine zipper ATF-like transcription factor (BATF), TCF4, and others (Fig. 1E and Table 1). By contrast, some members of bZIP and zinc finger (ZC) family were highly downregulated in *Fam20c*-deficient osteoblasts, including FRA1, ATF3, BATF, activator protein-1 (AP-1), FRA2, JUNB, fos-like antigen 2 (FOSL2), JUN-AP1, BTB and CNC homology 2 (BACH2), and CCCTC-binding factor (CTCF) (Fig. 1F and Table 2).

**Table 1:** Motif enrichment results up regulated in OB *Fam20c*^*KO*^ (top 20 displayed)

Motif Name	Consensus	P-value	% of Target Sequences with Motif	% of Background Sequences with Motif
Tcf21(bHLH)	NAACAGCTGG	1E−174	26.00%	12.06%
Fra2(bZIP)	GGATGACTCATC	1E−159	12.76%	3.94%
Fra1(bZIP)	NNATGASTCATH	1E−153	13.55%	4.49%
JunB(bZIP)	RATGASTCAT	1E−150	13.53%	4.54%
Atf3(bZIP)	DATGASTCATHN	1E−146	14.96%	5.48%
BATF(bZIP)	DATGASTCAT	1E−146	15.04%	5.53%
TCF4(bHLH)	SMCATCTGKH	1E−140	34.85%	20.23%
Fosl2(bZIP)	NATGASTCABNN	1E−137	10.01%	2.86%
Jun-AP1(bZIP)	GATGASTCATCN	1E−136	8.07%	1.92%
Atoh1(bHLH)	VNRVCAGCTGGY	1E−134	26.94%	14.16%
Ascl1(bHLH)	NNVVCAGCTGBN	1E−134	34.94%	20.58%
Ap4(bHLH)	NAHCAGCTGD	1E−132	28.71%	15.65%
AP-1(bZIP)	VTGACTCATC	1E−128	16.07%	6.63%
Tcf12(bHLH)	VCAGCTGYTG	1E−123	23.74%	12.18%
NeuroG2(bHLH)	ACCATCTGTT	1E−120	32.87%	19.50%
Ptf1a(bHLH)	ACAGCTGTTN	1E−119	49.90%	34.59%
HEB(bHLH)	VCAGCTGBNN	1E−111	38.56%	24.82%
Chop(bZIP)	ATTGCATCAT	1E−109	6.22%	1.42%
Atf4(bZIP)	MTGATGCAAT	1E−101	6.77%	1.79%
E2A(bHLH)	DNRCAGCTGY	1E−98	30.83%	18.95%

% of Target Sequence with motif: The ratio of the predicted transcription factor binding sites on a given DNA sequence to all transcription factor binding sites; % of Background Sequence with Motif: The ratio of the predicted transcription factor binding sites on the background sequence (genomic DNA sequence) to all transcription factor binding sites

**Table 2:** Motif enrichment results down regulated in OB *Fam20c*^*KO*^ (top 20 displayed)

Motif Name	Consensus	P-value	% of Target Sequences with Motif	% of Background Sequences with Motif
Fra1(bZIP)	NNATGASTCATH	1E−423	28.85%	5.48%
Atf3(bZIP)	DATGASTCATHN	1E−422	31.25%	6.56%
BATF(bZIP)	DATGASTCAT	1E−412	31.11%	6.67%
AP-1(bZIP)	VTGACTCATC	1E−400	32.08%	7.34%
Fra2(bZIP)	GGATGACTCATC	1E−396	25.71%	4.53%
JunB(bZIP)	RATGASTCAT	1E−396	27.88%	5.45%
Fosl2(bZIP)	NATGASTCABNN	1E−324	19.51%	3.05%
Jun-AP1(bZIP)	GATGASTCATCN	1E−285	15.71%	2.15%
CTCF(Zf)	AYAGTGCCMYCTRGTGGCCA	1E−124	7.06%	0.98%
Bach2(bZIP)	TGCTGAGTCA	1E−114	8.63%	1.68%
BORIS(Zf)	CNNBRGCGCCCCCTGSTGGC	1E−90	7.06%	1.41%
RUNX2(Runt)	NWAACCACADNN	1E−30	14.40%	8.42%
RUNX(Runt)	SAAACCACAG	1E−29	12.28%	6.90%
TEAD1(TEAD)	CYRCATTCCA	1E−27	15.80%	9.89%
RUNX-AML(Runt)	GCTGTGGTTW	1E−26	11.65%	6.70%
RUNX1(Runt)	AAACCACARM	1E−25	16.57%	10.71%
TEAD(TEA)	YCWGGAATGY	1E−24	11.71%	6.85%
MafK(bZIP)	GCTGASTCAGCA	1E−24	5.77%	2.55%
c-Jun-CRE(bZIP)	ATGACGTCATCY	1E−22	5.43%	2.42%
TEAD3(TEA)	TRCATTCCAG	1E−21	17.34%	11.81%

% of Target Sequence with motif: The ratio of the predicted transcription factor binding sites on a given DNA sequence to all transcription factor binding sites; % of Background Sequence with Motif: The ratio of the predicted transcription factor binding sites on the background sequence to all transcription factor binding sites

### Joint profiling of chromatin accessibility and gene expression

**Fig. 2:**
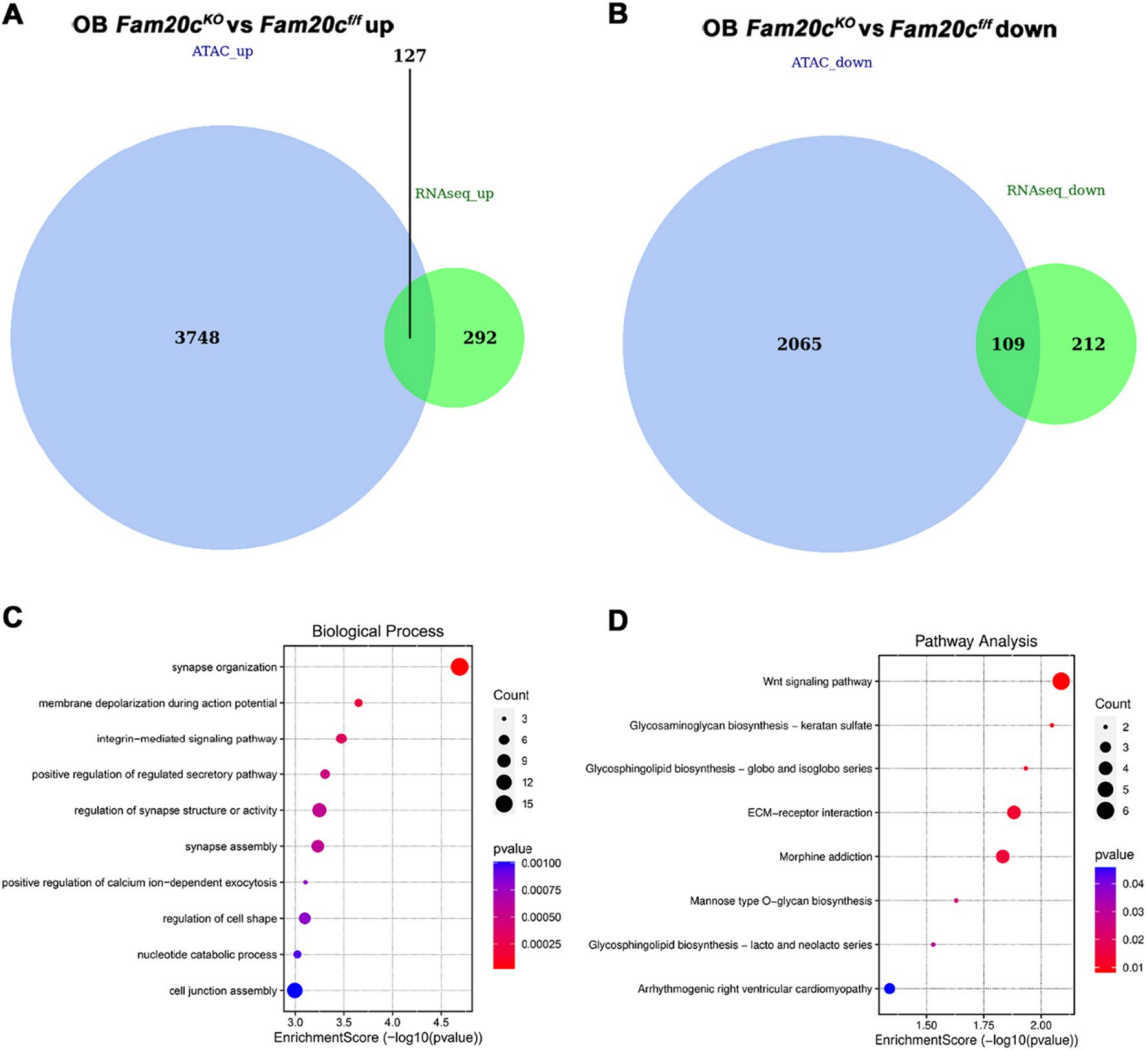
Analysis of ATAC-seq and intersection with RNA-seq in *Fam20c* deficient osteoblast. **A** Venn diagram depicts the intersection of genes with DAR-associated up-regulated and up-regulated DEGs in OB *Fam20c*^*KO*^. **B** Venn diagram depicts the intersection of genes with DAR-associated down-regulated and down-regulated DEGs in OB *Fam20c*^*KO*^. **C** Biological Processes of Gene Ontology (GO) enrichment analysis. The colors of circle dots illustrate the *P*-values identified for each GO term (low: red, high: blue), with lower values for more significant enrichment. The size of dots indicates the number of the differentially expressed genes and the larger dots represent a larger gene number. **D** Kyoto Encyclopedia of Genes and Genomes (KEGG) pathway enrichment analysis. The colors of circle dots illustrate the *P*-values identified for each GO term (low: red, high: blue), with lower values for more significant enrichment. The size of dots indicates the number of the differentially expressed genes and the larger dots represent a larger gene number

To create a chromatin accessibility and mRNA expression co-profiling analysis that is responsible for transcription factors regulation of downstream genes, we combined our ATAC-seq data with RNA-seq data. RNA-seq data was performed in our previous publication [28]. Detailed gene expression and Q value were summarized in Additional file 10: Table S3. The filter criteria for differentially expressed genes (DEGs) were as follows: log2 (Fold Change) > 1 or < −1 and Q value < 0.05. We first intersected related genes of DARs in ATAC-seq with DEGs of RNA-seq to obtain overlapping genes. Overall, OB *Fam20c*^*KO*^ versus OB *Fam20c*^*f/f*^ identified 3875 up-regulated genes nearest to DARs, and 127 overlapped with up-regulated DEGs (Fig. 2A and Additional file 11: Table S4). While we obtained 2174 down-regulated genes associated with DARs, 321 down-regulated DEGs, and the overlap part was 109 genes (Fig. 2B and Additional file 12: Table S5). We further assessed gene functions that may be regulated by transcription factors by performing GO and KEGG pathway analysis on the corresponding overlapping gene between ATAC-seq and RNA-seq. GO categories associated with *Fam20c*-deficient genes included synapse organization, membrane depolarization during action potential, integrin-mediated signaling pathway, regulation of synapse structure or activity, and regulation of cell shape, linking the observed chromatin alternated events to cellular shape and synapse changes (Fig. 2C and Additional file 13: Table S6). KEGG signal pathway analysis revealed enrichment for Wnt signaling pathway, glycosaminoglycan biosynthesis, and extracellular matrix (ECM)-receptor interaction (Fig. 2D and Additional file 13: Table S7).

### Proteomics characterization of *Fam20c*-deficient osteoblasts

**Fig. 3:**
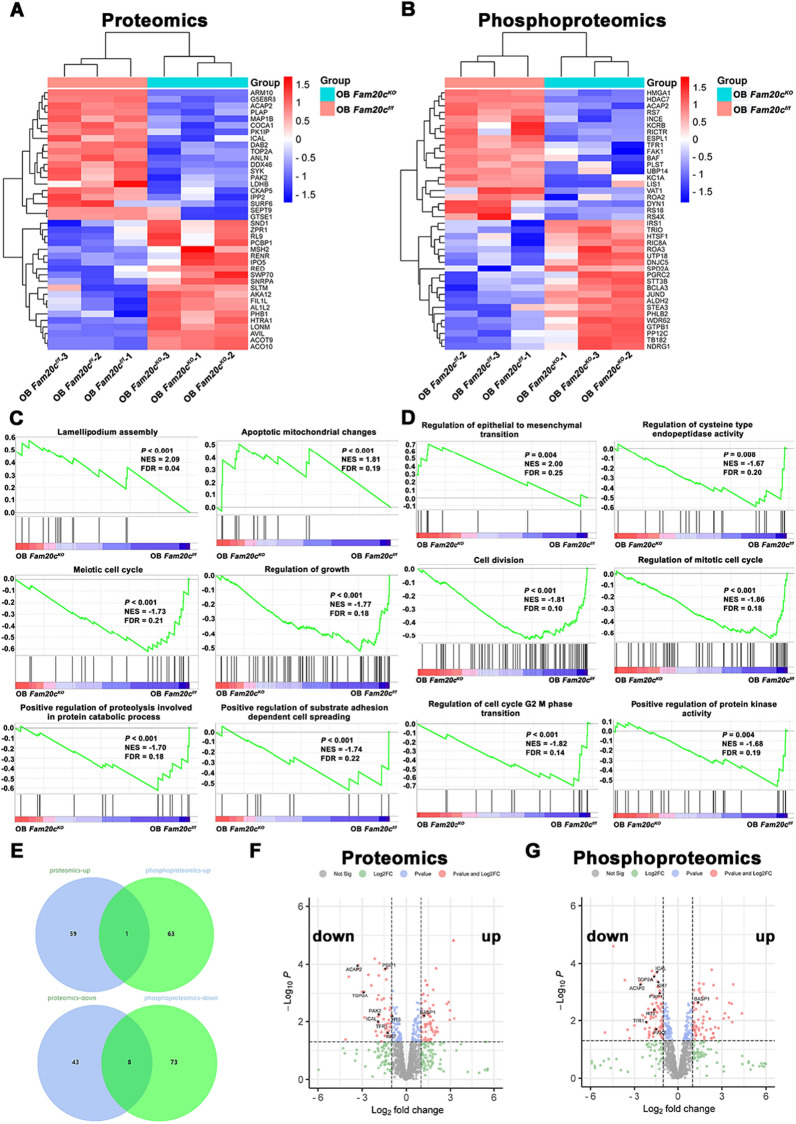
Analysis of Proteomics and Phosphoproteomics in *Fam20c* deficient osteoblast. **A** Clustered heatmap of top 40 (20 up-regulated and 20 down-regulated) differentially expressed proteins between OB *Fam20c*^*f/f*^ and OB *Fam20c*^*KO*^ in proteomics. **B** Clustered heatmap of top 40 (20 up-regulated and 20 down-regulated) differentially expressed proteins between OB *Fam20c*^*f/f*^ and OB *Fam20c*^*KO*^ in phosphoproteomics. **C** The enrichment of differentially expressed proteins between OB *Fam20c*^*f/f*^ and OB *Fam20c*^*KO*^ were analyzed via gene set enrichment analysis (GSEA). **D** The enrichment of differentially expressed phosphorylated proteins between OB *Fam20c*^*f/f*^ and OB *Fam20c*^*KO*^ were analyzed via gene set enrichment analysis (GSEA). **E** Venn diagram depicts the intersection of proteins with differentially expressed proteins and differentially expressed proteins. The upper diagram is the up-regulated part, below depicts down-regulated part. **F** Volcano plot of differentially expressed proteins based on proteomics data with 9 intersected proteins labeled. **G** Volcano plot of differentially expressed phosphorylated proteins based on phosphorylated proteomics data with 9 intersected proteins labeled

Next, we extended our analyses to identify changes that span transcription factors to RNA to protein in *Fam20c*-deficient osteoblasts. Proteomic data can better reflect gene function than transcriptomic data [39]. Using label-free quantitative proteomics, we quantified 8085 and 8146 peptides representing 4080 and 3874 proteins from OB *Fam20c*^*f/f*^ and OB *Fam20c*^*KO*^ with a false discovery rate (FDR) less than 1% at both peptide (Additional file 14: Table S8). We performed a heatmap analysis based on proteins differentially expressed, together, and identified 61 upregulated proteins and 51 downregulated proteins in the OB *Fam20c*^*KO*^ group (p-value < 0.05, log2Fold change > 1). The upregulated and downregulated top 20 proteins were shown in Fig. 3A. For the phosphoproteomic data, a total of 8453 and 8503 phosphopeptides representing 4504 and 4444 phosphoproteins were identified from OB *Fam20c*^*f/f*^ and OB *Fam20c*^*KO*^ (Additional file 15: Table S9). Difference analysis-based phosphoproteomic data revealed 64 upregulated and 80 downregulated phosphoproteins in OB *Fam20c*^*KO*^, with normalization by the z-score (p-value < 0.05, log2Fold change > 1). Figure 3B showed the top 20 phosphorylated proteins up- and down-regulated.

Our following step was to learn more about the signaling pathways that these proteins and phosphoproteins regulated the biological behaviors of OB *Fam20c*^*KO*^. GSEA on the expression profiles of proteomics and phosphoproteomics was performed. The proteins associated with lamellipodium assembly and apoptotic mitochondrial were strongly enriched in OB *Fam20c*^*KO*^ (Fig. 3C). OB *Fam20c*^*f/f*^ showed high amounts enriched in cell growth, division, spreading, and protein catabolic process (Fig. 3C). In addition, for phosphorylated proteins, depletion of *Fam20c* resulted in increasing enriched in epithelial to mesenchymal transition (EMT) (Fig. 3D), which was consistent with our previous bioinformatic analysis of Fam20C in pan-cancer [40]. Likewise, the phosphorylated proteins of OB *Fam20c*^*f/f*^ showed high amounts enriched in cell division, cell cycle, cysteine-type endopeptidase activity, and protein kinase activity (Fig. 3D). Altogether, the deficiency of cell proliferation, migration, and protein catabolic was possibly a feature of OB *Fam20c*^*KO*^ cell.

We attempted to identify key molecules from the proteome and phosphoproteome subgroups by combining the multi-omics data. Since there was a similar GSEA trend between proteomics and phosphorylated proteomics, we intersected the differential proteins in this two proteomics. As shown in Fig. 3E, only one protein expressed level and phosphorylated level were upregulated, while the expression and phosphorylated levels of 8 proteins were down-regulated in OB *Fam20c*^*KO*^. The volcano plots visualized these 9 intersected proteins (Fig. 3F, G). Noteworthy, these 9 intersected proteins contained Calpastatin (its protein entry was ICAL). This protein is an endogenous inhibitor of Calpain [41], and its phosphorylation state mediated the enzyme activity of Calpain has been reported [42]. Calpain is a calcium-dependent cysteine protease, and, importantly, it plays a key role in regulating cellular physiological processes, including proteolysis, cytoskeleton remodeling, cell cycle regulation, apoptosis, cell signal transduction, and others [43, 44]. Moreover, the biological processes regulated by Calpain was highly related to the results of the above GSEA.

Furthermore, the activation of Calpain could promote proteolysis, thus realizing negative regulation of the Wnt pathway [45], which was highly enriched in joint profiling between ATAC-seq and RNA-seq (Fig. 2D). We next examined Calpastatin expression and phosphorylation levels, as well as Calpain levels.

### *Fam20c* knockout influences Calpastatin/Calpain proteolysis system in vitro/vivo

**Fig. 4:**
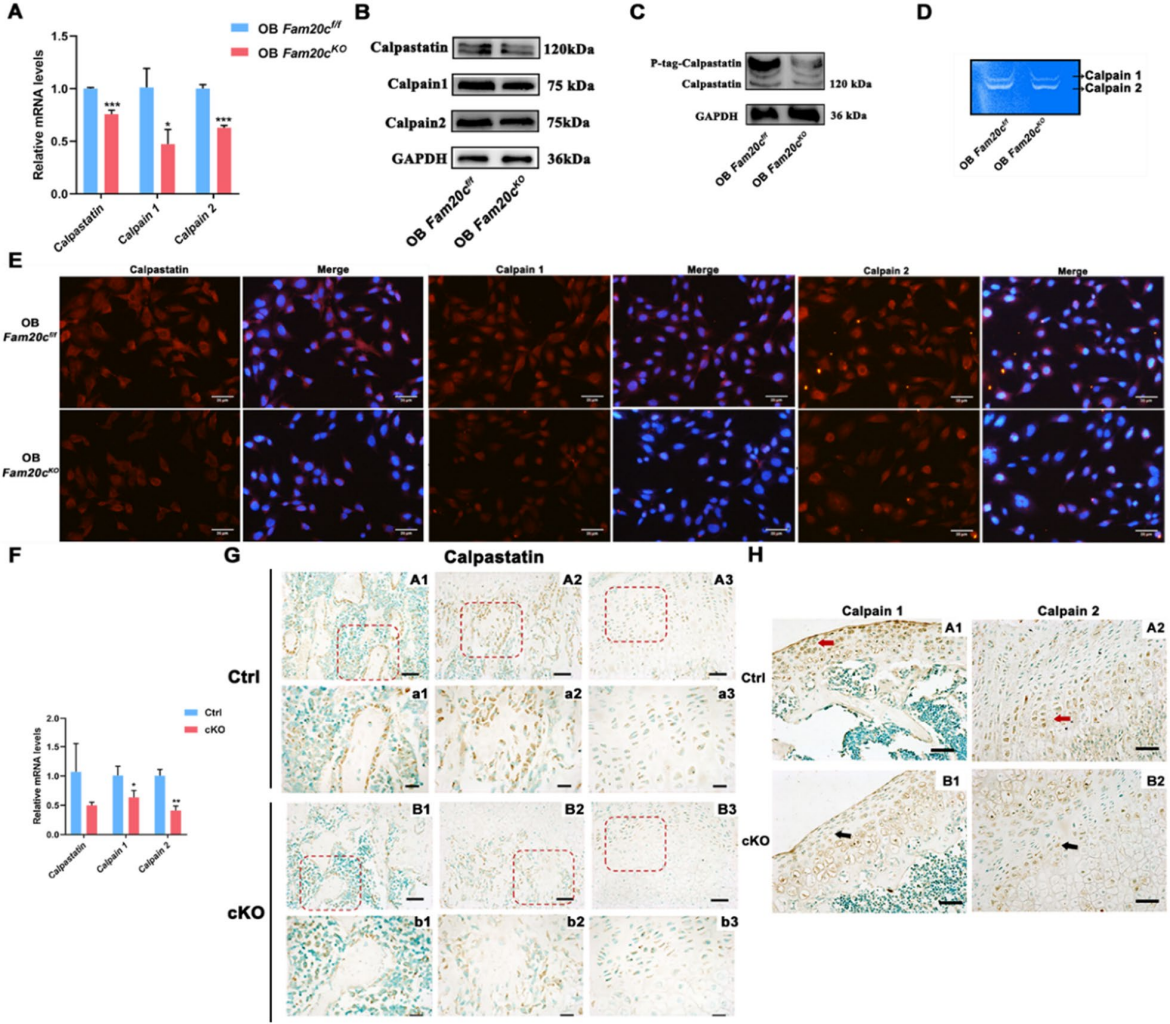
*Fam20c* affects the Calpastatin/Calpain proteolysis system. **A** Expression (qPCR) of *Calpastatin*, *Calpain 1,* and *Calpain 2* in OB *Fam20c*^*f/f*^ and OB *Fam20c*^*KO*^. ^*^P < 0.05, ^***^P < 0.001. **B** Western blot analysis of Calpastatin, Calpain 1, and Calpain 2 expression in OB *Fam20c*^*f/f*^ and OB *Fam20c*^*KO*^. GAPDH served as an internal control. **C** Phos-Tag sodium dodecyl sulfate–polyacrylamide gel electrophoresis (SDS-PAGE) (P-tag) demonstrates Calpastatin phosphorylation levels in OB *Fam20c*^*f/f*^ and OB *Fam20c*^*KO*^. GAPDH served as an internal control. **D** Casein zymography profiles of Calpain 1 and Calpain 2 in OB *Fam20c*^*f/f*^ and OB *Fam20c*^*KO*^. **E** Immunofluorescence staining for Calpastatin, Calpain 1, and Calpain 2 in OB *Fam20c*^*f/f*^ and OB *Fam20c*^*KO*^. Scale bar = 20 µm. **F** Gene expression of *Calpastatin*, *Calpain 1,* and *Calpain 2* in Ctrl and cKO mice femurs. ^*^P < 0.05, ^**^P < 0.01. **G** Immunohistochemical staining for Calpastatin in 4-week-old Ctrl and cKO mice femurs. (A1) and (B1) were free bone trabeculae in the bone marrow. (A2) and (B2) were the site of bone formation in the calcified cartilage. (A3) and (B3) were the junction of the cartilage proliferation area and hypertrophic area. Red box represented local magnification, scale bar = 50 μm. (a1), (a2), (a3), (b1), (b2), (b3) were the corresponding magnification regions, scale bar = 20 μm. **H** Immunohistochemical staining for Calpain 1 and Calpain 2 in 4-week-old Ctrl and cKO mice femurs. Scale bar = 50 μm

Calpastatin/Calpain proteolysis system emerged in our research as a potential major protein, thus, we examined and verified the key molecular expression and activity of proteolysis system. Among the known 15 kinds of Calpain, Calpain 1 and Calpain 2 are the most widely distributed and studied. qPCR, Western blot, and Immunofluorescence analyses showed that *Fam20c* knockout resulted in a decreased expression of Calpastatin in OB *Fam20c*^*KO*^ cells (Fig. 4A, B, E), whereas accompanied by a decreased phosphorylated expression level of Calpastatin (Fig. 4C). These were consistent with the results of proteomics and phosphoproteomics. Furthermore, *Fam20c*-deficient osteoblasts displayed a diminished expression of Calpain 1 and Calpain 2 (Fig. 4A, B, E) and attenuation of Calpain activity (Fig. 4D).

To validate our in vitro results, we used a conditional *Fam20c* knockout model (*Osx-Cre; Fam20c*^*flox/flox*^, abbreviated as “cKO mice” in this report) and matching control (*Fam20c*^*flox/flox*^, referred to as “Ctrl”). We isolated femurs of 4-week-old mice, removed bone marrow, and exacted mRNA from bone tissue. Analysis of the mRNA expression of *Calpastatin* in Ctrl versus cKO mice identified no significant change, whereas consistent with in vitro results, the gene expression of *Calpain 1* and *Calpain 2* decreased significantly (Fig. 4F). An immunohistochemistry (IHC) staining analysis displayed that Calpastatin immunoreactivity was low in preosteoblasts on trabecular bone surfaces and cartilage calcification area in cKO mice (Fig. 4G). No significant difference was found in the expression of cartilage proliferation area in cKO mice compared with Ctrl mice. Specifically, although there was less expression of Calpastatin in the hypertrophic cartilage area compared with the proliferation area in Ctrl mice, there was almost no expression in cKO mice. For the expression of Calpain 1 and Calpain 2, as anticipated, IHC staining showed a significant reduction in their immunoreactivity (Fig. 4H). Calpain 1 was mainly expressed in the articular surface cartilage proliferation layer (arrows A1 and B1), while the intensity of Calpain 2 expression was mainly in the cartilage proliferation layer (arrows A2 and B2). Hence, these data demonstrate that Calpain 1 and Calpain 2 might play a role in different locations.

### *Fam20c* knockout-related changes in migration velocity correlate with changes in the organization of the F-actin cytoskeleton

**Fig. 5:**
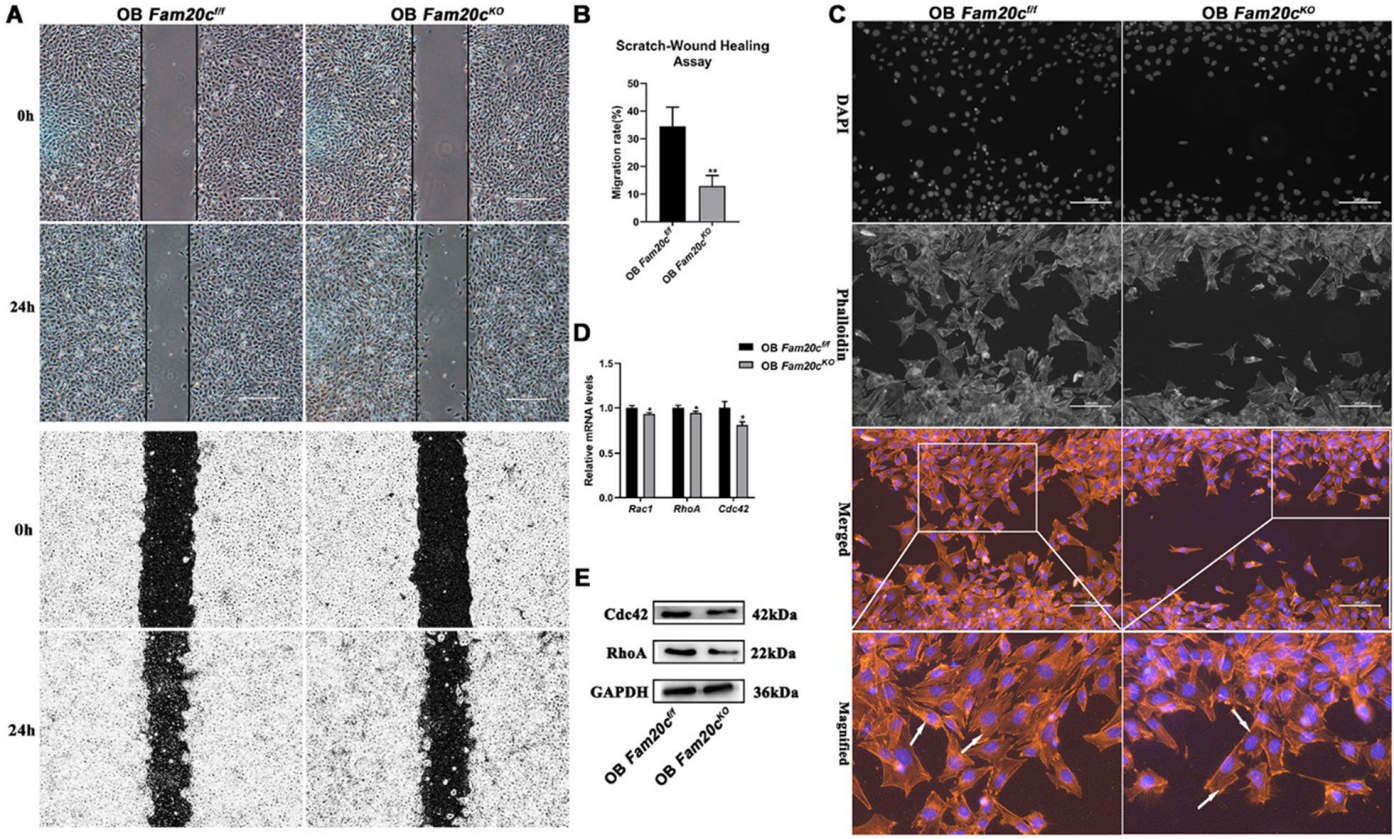
Migration and F-actin behavior in response to knockout of *Fam20c* in osteoblast. **A** Wounding healing assays of OB *Fam20c*^*f/f*^ and OB *Fam20c*^*KO*^, images were acquired 0 h and 24 h after the scratch. Scale bar = 100 μm. **B** Bar graph depicting cell migration rate (%) in a scratch wound-healing assay in OB *Fam20c*^*f/f*^ and OB *Fam20c*^*KO*^. ^**^P < 0.01, *t* test. **C** OB *Fam20c*^*f/f*^ and OB *Fam20c*^*KO*^ were stained by Phalloidin and DAPI to visualize F-actin and nucleus, respectively. OB *Fam20c*^*KO*^ showed a decreased amount of F-actin formation as compared with OB *Fam20c*^*f/f*^. White arrowheads indicate heavily polymerized F-actin. Scale bar = 100 µm. **D** Gene expression of *Rac1*, *RhoA*, and *Cdc42* in OB *Fam20c*^*f/f*^ and OB *Fam20c*^*KO*^. ^*^P < 0.05. **E** Western blot analysis of Cdc42 and RhoA expression in OB *Fam20c*^*f/f*^ and OB *Fam20c*^*KO*^. GAPDH served as an internal control

To determine if the weakened Calpastatin/Calpain proteolysis system seen in *Fam20c*-deficient cells and cKO mice would cause dysfunction. We measured cell proliferation and migration, based on the results of GSEA analysis of proteomics and phosphoproteomics and the biological process regulated by Calpastatin/Calpain proteolysis system as noted in previous studies (Fig. 3C, D) [43, 44]. OB *Fam20c*^*KO*^ showed less potent proliferation than control cells (Additional file 2: Figure S2). To dissect the role of FAM20C in osteoblast migration, we analyzed migration ability of OB *Fam20c*^*f/f*^ and OB *Fam20c*^*KO*^ by applying a monolayer scratch-wound assay in vitro. *Fam20c-*deficient cells displayed a relatively reduced cell migration (Fig. 5A). To confirm this, we quantified the scratch area at 0 h and 24 h applying Image J (MRI wound healing tool plugin), respectively, and through the formusla: (0 h scratch area -24 h scratch area)/ 0 h scratch area, to obtain migration rate. The results suggested that *Fam20c-*deficient cells had a lower migration rate, which was around 3 times slower than the OB *Fam20c*^*f/f*^ (Fig. 5B).

Coordinated actin morphology and cytoskeleton arrangement are key steps in migration [46, 47]. In this regard, Calpain proteases also participate mediate cytoskeleton events [48, 49]. To this end, we assessed the indicators of actin cytoskeleton arrangement in OB *Fam20c*^*f/f*^ and OB *Fam20c*^*KO*^. Using phalloidin staining for F-actin in a scratch wound assay, we observed the changes in the cytoskeleton of cells moving towards the site of wounding. Similarly, the loss of *Fam20c* was less adept in wound closure in cell migration (Fig. 5C). In addition, the differences in the F-actin filaments at the leading edge of the wound between OB *Fam20c*^*f/f*^ and OB *Fam20c*^*KO*^ are significant (Fig. 5C). At the leading edge of the scratch wound, OB *Fam20c*^*f/f*^ cells exhibited a highly elongated shape through the extension of the pseudopod-like structure, while OB *Fam20c*^*KO*^ cells adopt a square round in a relatively quiescent state. Furthermore, we found that the deletion of *Fam20c* contributed to decreased F-actin formation as indicated by the arrows in Fig. 5C, which was not observed in OB *Fam20c*^*f/f*^. These findings suggested that *Fam20c* may participate in the regulation of appropriate cellular actin morphology. Expression of the actin cytoskeleton organization regulators Ras homolog gene family member A (RhoA), Ras-related C3 botulinum toxin substrate 1 (Rac1), and Cell division cycle 42 (Cdc42) as measured by PCR and Western blot were decreased in OB *Fam20c*^*KO*^ compared with OB *Fam20c*^*f/f*^ (Fig. 5D, E).

### Impaired Calpastatin/Calpain proteolysis system in *Fam20c*-deficient cells is associated with weak mobility and F-actin formation disorder signature

**Fig. 6:**
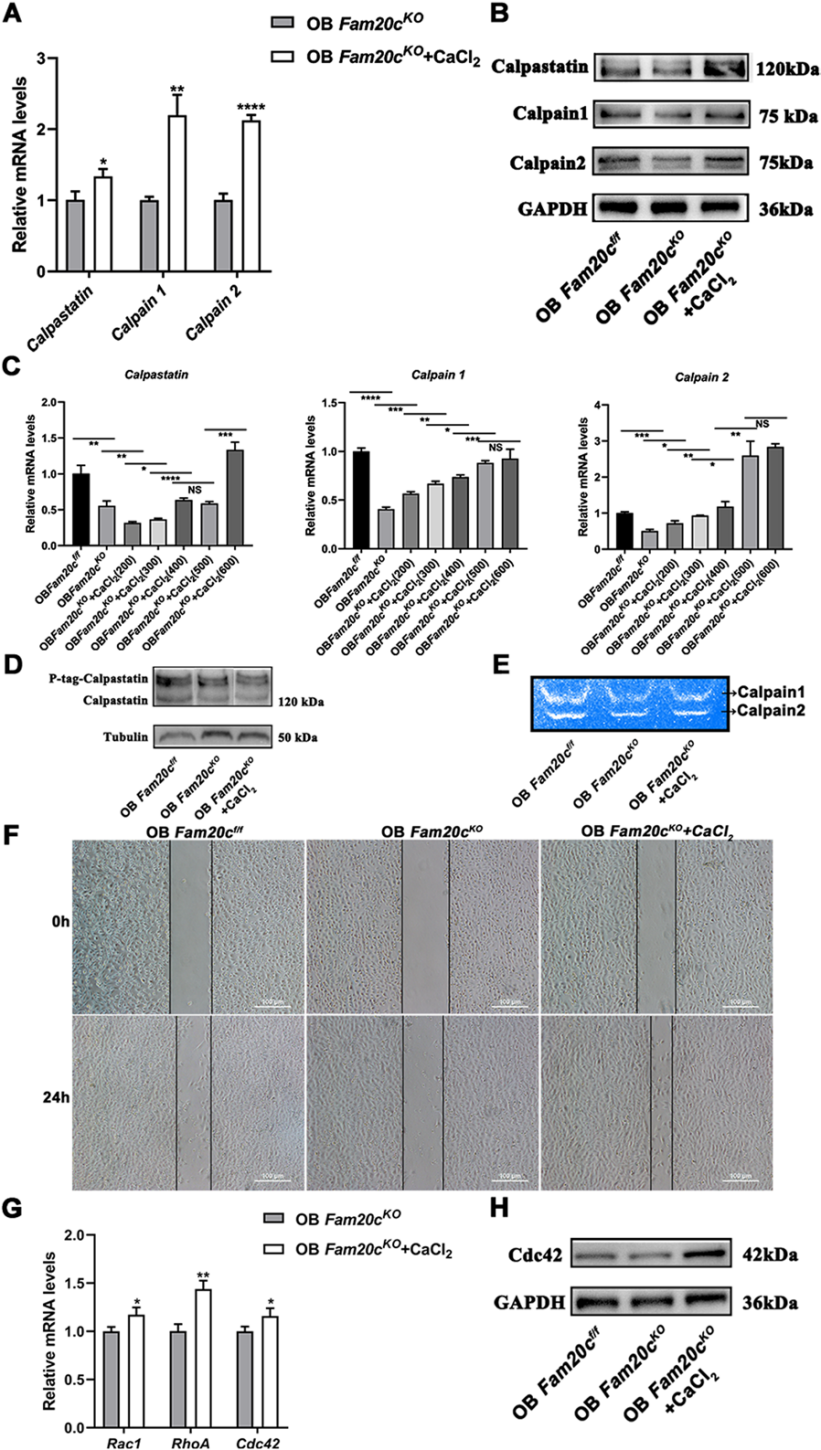
Calpastatin/Calpain proteolysis system influences cell migration velocity and F-actin behavior in *Fam20c*-deficient cells. **A** Gene expression of *Calpastatin*, *Calpain 1,* and *Calpain 2* in OB *Fam20c*^*KO*^ and OB *Fam20c*^*KO*^ treated with CaCl_2_. ^*^P < 0.05, ^**^P < 0.01, ^****^P < 0.0001. **B** Western blot analysis of Calpastatin, Calpain 1, and Calpain 2 expression in OB *Fam20c*^*KO*^ and OB *Fam20c*^*KO*^ treated with CaCl_2_. GAPDH served as an internal control. **C** Gene expression of *Calpastatin*, *Calpain 1*, and *Calpain 2* in OB *Fam20c*^*f/f*^, OB *Fam20c*^*KO*^, and OB *Fam20c*^*KO*^ treated with different concentrations of CaCl_2_. ^*^P < 0.05, ^**^P < 0.01, ^***^P < 0.001, ^****^P < 0.0001, NS: no significance. **D** Phos-Tag sodium dodecyl sulfate–polyacrylamide gel electrophoresis (SDS-PAGE) (P-tag) demonstrates effects of CaCl_2_ on Calpastatin phosphorylation levels in OB *Fam20c*^*f/f*^, OB *Fam20c*^*KO*^, and OB *Fam20c*^*KO*^ treated with CaCl_2_. Tubulin served as an internal control. **E** Casein zymography profiles of Calpain 1 and Calpain 2 in OB *Fam20c*^*f/f*^, OB *Fam20c*^*KO*^, and OB *Fam20c*^*KO*^ treated with CaCl_2_. **F** Wounding healing assays of OB *Fam20c*^*f/f*^, OB *Fam20c*^*KO*^, and OB *Fam20c*^*KO*^ treated with CaCl_2_, images were acquired 0 h and 24 h after the scratch. Scale bar = 100 μm. **G** Gene expression of *Rac1*, *RhoA*, and *Cdc42* in OB *Fam20c*^*KO*^ and OB *Fam20c*^*KO*^ treated with CaCl_2_. ^*^P < 0.05, ^**^P < 0.01. **H** Western blot analysis of Cdc42 expression in OB *Fam20c*^*f/f*^, OB *Fam20c*^*KO*^ and OB *Fam20c*^*KO*^ treated with CaCl_2_. GAPDH served as an internal control

To further link the weak mobility phenotype in OB *Fam20c*^*KO*^ to the impaired Calpastatin/Calpain proteolysis system, we treated OB *Fam20c*^*KO*^ with low doses of Calcium chloride (CaCl_2_) (600 μmol/L) and repeated the above experiments. The concentration of CaCl_2_ was determined by the CCK8 experiment, the detailed information was seen in Additional file 3: Figure S3. In comparison with OB *Fam20c*^*f/f*^, OB *Fam20c*^*KO*^ treated with CaCl_2_ showed significantly increased expression of Calpastatin, Calpain 1, and Calpain 2 (Fig. 6A, B). Of note, the gene expression of *Calpain 1* and Calpain 2 exhibited a concentration-dependent elevation with the addition of CaCl_2_ in OB *Fam20c*^*KO*^ (Fig. 6C). No significant phosphorylated expression level of Calpastatin difference was observed in OB *Fam20c*^*KO*^ with or without CaCl_2_ (Fig. 6D), whereas OB *Fam20c*^*KO*^ with CaCl_2_ showed higher Calpain activity (Fig. 6E). This treatment rescued almost entirely the deficit of OB *Fam20c*^*KO*^ in migrating (Fig. 6F). In view of the results presented above, we further used PCR and Western blot assays to detect the expression of *Rac1*, *RhoA,* and *Cdc42*. Corroborating our predictions, there was an elevated expression in OB *Fam20c*^*KO*^ treated with or without CaCl_2_ (Fig. 6G, H).

### Changes of Wnt signal pathway in *Fam20c*-deficient cells

**Fig. 7:**
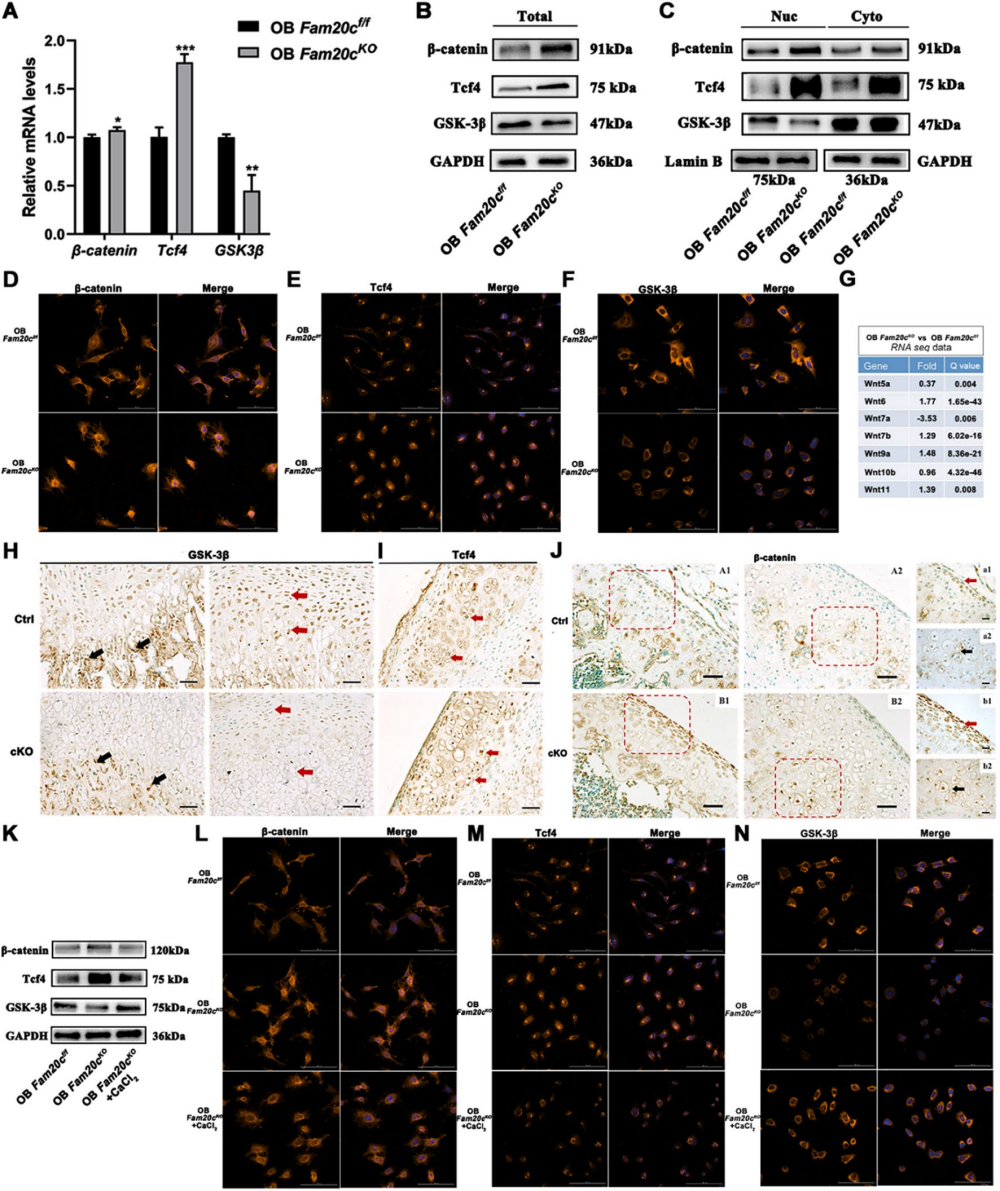
*Fam20c* affects the Wnt signaling pathway. **A** Gene expression of *β-catenin*, *Tcf4,* and *GSK-3β* in OB *Fam20c*^*f/f*^ and OB *Fam20c*^*KO*^. ^*^P < 0.05, ^**^P < 0.01, ^***^P < 0.001. **B** Western blot analysis of β-catenin, Tcf4, and GSK-3β expression in OB *Fam20c*^*f/f*^ and OB *Fam20c*^*KO*^. GAPDH served as an internal control. **C** Western blot analysis of β-catenin, Tcf4, and GSK-3β of nuclear and cytoplasm expression in OB *Fam20c*^*f/f*^ and OB *Fam20c*^*KO*^. GAPDH served as an internal control. **D** Immunofluorescence staining for β-catenin in OB *Fam20c*^*f/f*^ and OB *Fam20c*^*KO*^. Scale bar = 100 µm. **E** Immunofluorescence staining for Tcf4 in OB *Fam20c*^*f/f*^ and OB *Fam20c*^*KO*^. Scale bar = 100 µm. **F** Immunofluorescence staining for GSK-3β in OB *Fam20c*^*f/f*^ and OB *Fam20c*^*KO*^. Scale bar = 100 µm. **G** RNA-seq data in OB *Fam20c*^*f/f*^ and OB *Fam20c*^*KO*^. Fold represents log2fold change. **H** Immunohistochemical staining for GSK-3β in 4-week-old Ctrl and cKO mice femurs. The black arrows showed the area where cartilage calcifies to form bone trabecular, and the red arrows indicated a hypertrophic layer of cartilage. Scale bar = 50 μm. **I** Immunohistochemical staining for Tcf4 in 4-week-old Ctrl and cKO mice femurs. Scale bar = 50 μm. **J** Immunohistochemical staining for β-catenin in 4-week-old Ctrl and cKO mice femurs. (A1) and (B1) were proliferating layers of articular cartilage of the femur. (A2) and (B2) were the site of the hypertrophic cartilage layer on the surface of a joint. Red box represented local magnification, (A1), (A2), (B1), (B2), scale bar = 50 μm. (a1), (a2), (b1), (b2), were the corresponding magnification regions, scale bar = 20 μm. **K** Western blot analysis of β-catenin, Tcf4, and GSK-3β expression in OB *Fam20c*^*f/f*^, OB *Fam20c*^*KO*^, and OB *Fam20c*^*KO*^ treated with CaCl_2_. GAPDH served as an internal control. **L** Immunofluorescence staining for β-catenin in OB *Fam20c*^*f/f*^, OB *Fam20c*^*KO*^, and OB *Fam20c*^*KO*^ treated with CaCl_2_. Scale bar = 100 µm. **M** Immunofluorescence staining for Tcf4 in OB *Fam20c*^*f/f*^, OB *Fam20c*^*KO*^, and OB *Fam20c*^*KO*^ treated with CaCl_2_. Scale bar = 100 µm. **N** Immunofluorescence staining for GSK-3β in OB *Fam20c*^*f/f*^, OB *Fam20c*^*KO*^, and OB *Fam20c*^*KO*^ treated with CaCl_2_. Scale bar = 100 µm

The chromatin accessibility signature of OB *Fam20c*^*KO*^ suggested an enrichment of TCF4 motif (Fig. 1E), and *Fam20c*-deficient associated genes enrichment of Wnt signaling pathway was specifically observed in the joint profiling of ATAC-seq and RNA-seq data (Fig. 2D). Additionally, the depletion of *Fam20c* significantly changed Calpastatin/Calpain proteolysis system, as indicated by proteomics and phosphorylated proteomics analysis (Fig. 4). Importantly, previous studies have implicated Wnt signaling pathway could be regulated by Calpain activation to promote β-catenin degradation [45, 50]. We, therefore, set out to verify the expression of key molecules of Wnt signaling pathway in the subsequent experiments. RT-PCR revealed that *β-catenin* and *Tcf4* mRNA becomes prominently expressed in OB *Fam20c*^*KO*^, and *glycogen synthase kinase-3* (*GSK-3β*) was expressed at low levels (Fig. 7A). Consistent with the PCR results, protein levels displayed a similar trend (Fig. 7B). To confirm whether these changes mediated canonical Wnt signaling regulates nuclear translocation, we observed in OB *Fam20c*^*KO*^ that Tcf4 and β-catenin were mainly localized in the nuclear, and a decreased expression of GSK-3β was detected (Fig. 7C–F). Among the Wnt family, RNA-seq analysis revealed the mRNA level of canonical Wnt ligands, *Wnt family member 7a (Wnt7a)*, was suppressed (Fig. 7G). And other canonical Wnt ligands were not detected in the RNA-seq data. These data suggest that *Fam20c* activates Wnt/β-catenin signaling through *β-catenin* and *Tcf4* transcription and translation. To extend this observation in vivo, we analyzed these protein expressions from Ctrl and cKO mice. During bone formation, GSK-3β was strongly expressed in the area where cartilage calcified to form bone trabecular in Ctrl mice, and a decrease was observed in the cKO mice (Fig. 7H). Furthermore, compared with Ctrl mice, Tcf4 was highly accumulated in the nucleus of chondrocytes with an obvious elevated expression level in cKO mice (Fig. 7I). The role of β-catenin was also measured in vivo, ~ 90% of the nuclei displayed increased expression of Tcf4 in the proliferating layer of articular cartilage of the femur and hypertrophic cartilaginous layer of the articular surface (Fig. 7J).

We then investigated whether the Wnt signaling pathway is a target of Calpastatin/Calpain proteolysis system. After the treatment of CaCl_2_, we observed reduced Tcf4 and β-catenin and elevated GSK-3β in OB *Fam20c*^*KO*^, immunofluorescence further documented a decreased location of Tcf4 and β-catenin in OB *Fam20c*^*KO*^ nuclei (Fig. 7K–N).

## Discussion

Increasing evidence has pointed to essential roles for FAM20C phosphorylated substrates within S-x-E/pS motifs in regulating many physiological processes [1, 7, 51]. Despite this intrigue, the knowledge about FAM20C has remained in the infant stage resulting from its complex regulatory mechanisms. The widespread multiple FAM20C substrates, ranging from the secreted proteins to intracellular proteins, of which phosphorylated levels are altered [52, 53]. Accordingly, the complex regulatory network by FAM20C, including epigenetics, gene expression, and protein interactions, is attracting broad attention. Comprehensive multi-omics analysis could broaden our knowledge of the molecular events relevant to biological processes [54, 55]. Here, we hypothesized that the joint profiling analysis of chromatin accessibility, gene expression, proteomics, and phosphorylated proteomics data between OB *Fam20c*^*f/f*^ and OB *Fam20c*^*KO*^, may capture some new regulatory mechanism of FAM20C.

ATAC-seq allows the identification of genomic regions associated with gene-regulatory activity, thus providing a method to infer transcription factor (TF) activity [37]. Our data demonstrated that lack of *Fam20c* generally intensified 12% of the accessible regions in osteoblasts, leading to a global activation of downstream TFs (Fig. 1B). Motif analysis illuminated that TCF4 was highly enriched in the chromatin opened of *Fam20c* deficient osteoblasts (Fig. 1E). TCF4, a member belonging to the bHLH TF family, was regarded to play a vital role in many developmental processes [56, 57]. Furthermore, when Wnt ligands bind to the Frizzled-Lrp5/6 receptor complex, β-catenin degradation is blocked and translocated to the nucleus, allowing TCF to bind and to activate Wnt target genes [58, 59]. Consistently, we also found a significant enrichment of the KEGG pathway in the Wnt signaling pathway through joint profiling ATAC-seq and RNA-seq (Fig. 2D). Previous studies have shown that tissue homeostasis and cell maintenance could be impacted by Wnt signaling pathway [60, 61]. In this research, PCR, Western blot, and Immunofluorescence both elucidated that *Fam20c* knockout significantly activated the transcriptional activity of *β-catenin* and *Tcf4* signaling, as well as elevated the protein expression levels of them, especially, the increased expression levels in the nucleus. While GSK-3β displayed a contrary trend, that is, the total expression level and the nuclear expression level decreased (Fig. 7A–F).

Moreover, Qin’s research found that TCF1 and LEF1 were down-regulated in the vertebrae of *Sox2-Cre*; *Fam20c*^*foxl/flox*^ mice [62]. Further, TCF4 is combined with *β-catenin* upstream of BMP. In our research, knockout of *Fam20c* in osteoblasts in vivo, changes were only found in the hypertrophic cartilage layer, indicating that there were differences in vivo and in vitro, which might be caused by the paracrine and juxtacrine functions of cells. In addition, cartilage thickening has been observed in several condition knockout animal models, whether this phenomenon was related to the Wnt signaling pathway remains to be further investigated. Furthermore, studies have shown that Wnt signal can inhibit the expression of activated Col2a1, and the overexpression of *β-catenin* in chondrocytes can cause serious congenital chondrosis, indicating that its overexpression affects the bone development process, supporting the results of our study [63]. Therefore, we believe that after *Osx-Cre* knockout of *Fam20c*, the expression of downstream GSK-3β in the cytoplasm is inhibited, thus increasing the entry of *β-catenin* into the nucleus, and the overexpression of *β-catenin* in the nucleus overactivated TCF4. Finally, the proliferation of chondrocytes is induced through the Wnt signaling pathway, thus affecting the process of bone development.

In the current study, extensive Mass Spectrometry (MS)-based proteomics and phosphoproteomics analysis permitted in-depth screening of the changes in protein expression, post-translational phosphorylated modification, and biological processes alteration in *Fam20c* deficient osteoblasts. These included but were not limited to: (I) weakened changes in the cell growth, where the predominant changes were the regulation of mitotic cell cycle, cell division, and regulation of cell cycle G2 M phase transition; (II) a decrease in alteration in regulation of proteolysis involved in protein catabolic process; (III) alteration in cell migration (Fig. 3C, D). The deficiency of cell growth in OB *Fam20c*^*KO*^ was proved in previous studies [18, 24]. Of note, in this research, we verified the major driver protein was Calpastatin, which was less expressed and decreased phosphorylated levels in OB *Fam20c*^*KO*^ than in OB *Fam20c*^*f/f*^ (Fig. 4). We also performed experiments to knock out FAM20C in human 293 T cells and obtained a similar trend (Additional file 6: Figure S6). Calpastatin belongs to the calpain system; mediates cell motility, cell cycle, signal pathways transduction, apoptosis, and regulates gene expression via proteolytic degradation [43, 64, 65]. The other two members of the calpain system, Calpain 1 and Calpain 2, activity was mediated by Calpastatin phosphorylation state [42, 66, 67]. Specifically, the phosphorylated Calpastatin presented relatively static, located near the cell nucleus, and presented an aggregation state, that is, the activity of Calpastatin was inhibited, thus enhancing the activity of Calpain. On the contrary, the dephosphorylation of Calpastatin made its distribution in cells relatively favorable, and the activity of Calpastatin increased, which then weakens the activity of Calpain. Consistently, we observed *Fam20c*-deficient osteoblasts displayed a diminished expression of Calpain 1 and Calpain 2, and attenuation of Calpain activity (Fig. 4D). OB *Fam20c*^*KO*^ cells under the influence of impaired Calpastatin/Calpain proteolysis system exhibited weak mobility and guide differential remodeling of cytoskeletons at the leading edge versus the OB *Fam20c*^*f/f*^, with the inhibition of RhoA, Rac1, and Cdc42 (Fig. 5). The Rho family of small guanosine triphosphatases (GTPases), particularly RhoA, Rac1, and Cdc42, play essential roles in regulating the cell cycle and actin cytoskeleton, affecting cell adhesion and migration [68–70]. For cell migration, specifically, RhoA participated in the assembly of stress fibers, Rac1 was responsible for the formation of lamellar pseudopods, and Cdc42 regulated the shape of filamentous pseudopods [71, 72]. Moreover, we observed the weakened mobility was rescued by CaCl_2_ in OB *Fam20c*^*KO*^, accompanied by a concentration-dependent elevation of Calpain 1 and Calpain 2 expression and activity but no significant changes in Calpastatin phosphorylation level (Fig. 6), further indicating that cells migration barriers were associated with Calpastatin/Calpain proteolysis system. Calpain could regulate a variety of signaling pathways through proteolysis of target proteins, including cell cycle (cyclin D1 and cyclin E), cell survival (nuclear factor- κ B), and apoptosis (Bcl2 family and caspases) [73]. It was also possible that Calpain mediated cell migration through multiple mechanisms, as noted in previous studies, including the cleavage of integrin-related complex, adhesive plaque, Focal adhesion kinase (FAK), Paxillin, Talin, and other components, resulting in reduced cell adhesion and increased mobility [74, 75]. Similar to our in vitro results, we observed the expression of Calpastatin, Calpain 1, and Calpain 2 in the growth plate region in animal models at the time when articular cartilage changes were most visible (i.e. 4 weeks after birth), and the results were consistent with cytological trends. In the knockout group, the expression of Calpastatin in the calcified area of growth plate cartilage, the pre-osteoblasts on the surface of bone trabeculae and hypertrophic cartilage decreased significantly. Calpain 1 and Calpain 2 were expressed in different positions in the cartilage, among which Calpain 1 was mainly expressed in the surface and middle layers of articular cartilage, and Calpain 2 was mainly expressed in the deep layer. The expressions of both decreased significantly in the knockout group. Real-time quantitative PCR detection showed that the expression levels of *Calpain 1* and *Calpain 2* genes in bone tissue decreased. These results further prove that the Calpastatin/Calpain proteolysis system is inhibited overall after the conditional knockout of *Fam20c*, and the dysfunction of this system can cause abnormalities in its downstream signaling pathway, inhibit the functions of pre-osteoblasts and hypertrophic chondrocytes, and ultimately affect the process of cartilage osteogenesis and cancellous bone formation.

Regarding Calpastatin/Calpain proteolysis system’s influence on Wnt signaling pathway, we took note of some previous studies showing more than 100 substrates were downstream of Calpain, including β-catenin and GSK-3β of Wnt signaling pathway [76]. Briefly, Calpain activity could regulate β-catenin expression level, intracellular localization, and function [77].

The down-regulated of Calpain 2 expression, resulted in elevated β-catenin expression and accumulate in the nucleus. Conversely, Calpain activation could promote β-catenin degradation to achieve negative regulation of Wnt signaling pathway [45]. Lade et al. [50] reported that Calpain 1 induced N-terminal truncation of β-catenin in the mouse liver development process, this might play a vital role in regulating the differentiation of hepatoblasts. In addition, Goni-Oliver and Ma et al. [78, 79] reported that Calpain truncated C- and N-terminal self-suppressing domains of GSK-3β to activate GSK-3β. In this regard, we added CaCl_2_ to OB *Fam20c*^*KO*^ with weak calpain activity to activate calpain activity, and then the cell migration ability was enhanced, β-catenin and Tcf4 expression levels decreased, a decreased location of Tcf4 and β-catenin in OB *Fam20c*^*KO*^ nuclei (Fig. 7K–N). Another key to note in our study is that previous studies have indicated that activation of Wnt signaling pathway could promote cell proliferation and migration [80, 81]. However, activating Wnt signaling pathway had less pronounced benefits, we do not detect accelerated cell migration in OB *Fam20c*^*KO*^. Given that we found *Fam20c* depletion to cause a weakened Calpastatin/Calpain proteolysis system, which could promote the hydrolysis of migration-related proteins to accelerate cell migration and negatively manage the Wnt signaling pathway. Thus, in the absence of *Fam20c*, this phenomenon could be explained by the possible explanation that Calpastatin/Calpain proteolysis system acts predominantly in regulating cell migration.

Additional analysis of the Wnt ligands, as shown here, only *Wnt7a* was observed significant changes in canonical Wnt ligands (Fig. 7G). The overlapped genes obtained from ATAC-seq and RNA-seq were projected into the KEGG pathway and visualized, which also showed the enrichment of *Wnt7a* (Additional file 4: Figure S4). Several studies have highlighted the importance of Wnt7a in regulating cell migration and invasion [82, 83], however, its role was cell-specific. Xie et al. reported that Wnt7a promoted oral squamous cell carcinoma cell migration induced by epidermal growth factor via the activation of the β-catenin/Matrix metalloproteinase-9 (MMP-9) signal pathway [83]. Nevertheless, Lan et al. proposed the overexpression of Wnt7a inhibited the growth and migration of hepatocellular carcinoma in β-catenin-independent manner [84]. Taken together, our findings supported *Fam20c* depletion led to Wnt ligands down-regulated in osteoblasts, however, β-catenin increasingly translocated to the nucleus, triggering Wnt signaling pathway ligands-independent manner.

Furthermore, a series of elegant papers have reported that FAM20C could phosphorylate bone morphogenetic protein 4 (BMP4), while BMP2 and BMP7 might be possible substrates of FAM20C due to possessing S-x-E/pS motifs [7, 85]. Intriguingly, we have previously observed that in *Fam20c* salivary gland conditional knockout mice, the transcriptional and protein levels of BMP2 and BMP7 were up-regulated, while protein expression of BMP4 was reduced but did not change its transcript level [86]. The distribution pattern of BMP4 expression in salivary glands was almost not observed in the extracellular matrix, but concentrated in the cytoplasm, indicating that knockout of *Fam20c* caused abnormal secretion of BMP4. Also, Liu et al. showed mRNA levels of BMP2 and BMP7 were similarly increased in OB *Fam20c*^*KO*^, however, their downstream remains in a repressed state, implying that phosphorylation may affect the activity of BMP ligands [18]. In addition, as illustrated by our analyses, *Fam20c* depletion induced Calpastatin dephosphorylation, decreased Calpain activity, elevated β-catenin expression levels, and translocated into nucleus to combine with Tcf4, leading to the activation of Wnt signaling pathway in a ligands-independent manner. Since Wnt signaling pathway regulated the transcription of BMP [87, 88], thus, our findings provided a possibility to explain the enhanced transcription of BMP in *Fam20c*-deficient animal and cell models. On the other hand, increased transcription of BMP, but not activation of BMP signaling pathway, indicating that conduction inhibition occurred in BMP signaling pathway.

Finally, several prior studies have shown the loss of bone maturation phenotype in *Fam20c* cKO mice and loss-of-function of the in vivo gained osteoblasts [11, 15, 89]. Also, the absence of *Fam20c* distorted cell biological processes. Osteogenic differentiation blockade occurs in OB *Fam20c*^*KO*^, which could not be rescued by the addition of extracellular matrix proteins extracted from normal bone tissue, suggesting that FAM20C might regulate cell behaviors in a cell-autonomous manner [18]. Another characteristic of OB *Fam20c*^*KO*^, as above elucidated, was their paucity of BMP signaling pathway activation even with the elevated BMP ligands transcription, indicating that there was conduction abnormality in BMP signal pathway. Our integrated analysis revealed alterations of Calpastatin/Calpain proteolysis system and Wnt signaling pathway among the most dramatic differences between OB *Fam20c*^*f/f*^ and OB *Fam20c*^*KO*^. The aberrant Calpastatin/Calpain proteolysis system could alter the cleavage of specific proteins, regulating pathological and normal physiological processes that are thought to play critical roles in governing homeostasis [90, 91]. Also, the coordination of cell fates and tissue homeostasis is controlled by Wnt signaling pathway that regulates cell-to-cell communication [92, 93]. Then again, we found the signaling pathways related to cell signal transduction were significantly influenced in OB *Fam20c*^*KO*^, including mitogen-activated protein kinases (MAPK) signaling pathway, Ras signaling pathway, mammalian target of rapamycin (mTOR) signaling pathway, cAMP signaling pathway, etc. [24] (Additional file 5: Figure S5). These data provide experimental support for a paradigm in which OB *Fam20c*^*KO*^ appears the phenomenon of cellular homeostasis imbalance. Homeostasis is the basis of maintaining all kinds of important functions of the body, which involves tissue, cell, signal pathway, molecule, and others, breaking cell homeostasis will cause various pathological processes [94, 95]. These results comprise that the homeostatic imbalance of FAM20C knockout osteoblasts may involve changes in multiple signaling pathways in the conduction system.

Taken together, our current work provides an integrated and comprehensive analysis of FAM20C using multiple omics, including ATAC-seq, RNA-seq, proteomics, and phosphoproteomics. We revealed that Calpastatin/Calpain proteolysis system and Wnt signaling pathway alterations were possibly the key factors between OB *Fam20c*^*f/f*^ and OB *Fam20c*^*KO*^. Targeting Calpastatin phosphorylation may provide a promising research direction to study the role of FAM20C in development. However, this research still existed restrictions. Initially, the protein–protein interaction between FAM20C and Calpastatin needs to be examined in follow-up studies. In the future experiment, we will add FAM20C recombinant protein to OB *Fam20c*^*KO*^ to observe the change of phosphorylation of Calpastatin. Applying the radiolabeled technique to test the phosphorylation site of Calpastatin, constructing a mutant vector of the Calpastatin site to transfect OB *Fam20c*^*KO*^ to activate the phosphorylation of Calpastatin, and observing the expression and activity of Calpastatin/Calpain proteolytic system and its effect on osteoblast function. Second, we performed phosphorylated proteomics, and detected the difference in Calpastatin phosphorylation and the changes in the phosphorylated site after *Fam20c* gene knockout. Nevertheless, the exact phosphorylated site of Calpastatin has not been verified. Third, in the present study, we found the Calpastatin/Calpain proteolysis system could negatively regulate the Wnt signaling pathway. Further studies are needed to conduct experiments associated with Calpain activators/ inhibitors as well as activators/antagonists of the Wnt pathway to further define this regulatory relationship.

## Supplementary Information

**Additional file 1.** **Fig. S1**. Quality control for ATAC-seq samples generated in this study.

**Additional file 2.** **Fig. S2**. Cell proliferation assay of OB *Fam20c*^f/f^ and OB *Fam20c*^KO^ vitro.

**Additional file 3.** **Fig. S3** Cell proliferation assay and cytotoxicity assay by cell counting kit-8 in *Fam20c*^KO^ treated with CaCl_2_.

**Additional file 4.** **Fig. S4** Pathway map of overlapped genes from ATAC-seq and RNA-seq.

**Additional file 5.** **Fig. S5** Kyoto Encyclopedia of Genes and Genomes (KEGG) pathway enrichment analysis based on ATAC-seq.

**Additional file 6.** **Fig. S6** The changes of Calpastatin/Calpain proteolysis system in human 293T cells after *Fam20c* knock out

**Additional file 7.** **Table S1** ATAC-seq sequencing data quality control statistics.

**Additional file 8.** **Table S2** Statistics of the alignment results of Reads on the reference genome sequence.

**Additional file 9.** **Table S3** Gene expression and Q value of RNA-seq.

**Additional file 10.** **Table S4** 127 overlapped up-regulated differentially expressed genes between ATAC-seq and RNA-seq.

**Additional file 11.** **Table S5** 109 overlapped down-regulated differentially expressed genes between ATAC-seq and RNA-seq.

**Additional file 12.** **Table S6** Biological Processes of Gene Ontology (GO) enrichment analysis based on the corresponding overlapping gene between ATAC-seq and RNA-seq.

**Additional file 13.** **Table S7** Kyoto Encyclopedia of Genes and Genomes (KEGG) pathway enrichment analysis based on the corresponding overlapping gene between ATAC-seq and RNA-seq.

**Additional file 14.** **Table S8** The detailed information of differentially expressed peptides and proteins between OB *Fam20c*^f/f^ and OB *Fam20c*^KO^ in proteomics.

**Additional file 15.** **Table S9** The detailed information of differentially expressed phosphorylated peptides and proteins between OB *Fam20c*^f/f^ and OB *Fam20c*^KO^ in phosphoproteomics.

## Data Availability

Any data and R script in this study can be obtained from the corresponding author upon reasonable request. The final manuscript was read and approved by all authors.
