# Supplementary material for: Fam20c regulates the calpain proteolysis system through phosphorylating Calpasatatin to maintain cell homeostasis
Source: J Transl Med. 2023 Jun 27;21:417. doi: 10.1186/s12967-023-04275-4 (PMC10294482; doi:10.1186/s12967-023-04275-4)
Supplement: Supplementary file 1 — Additional file 1. Fig. S1. Quality control for ATAC-seq samples generated in this study. [file 12967_2023_4275_MOESM1_ESM.docx]

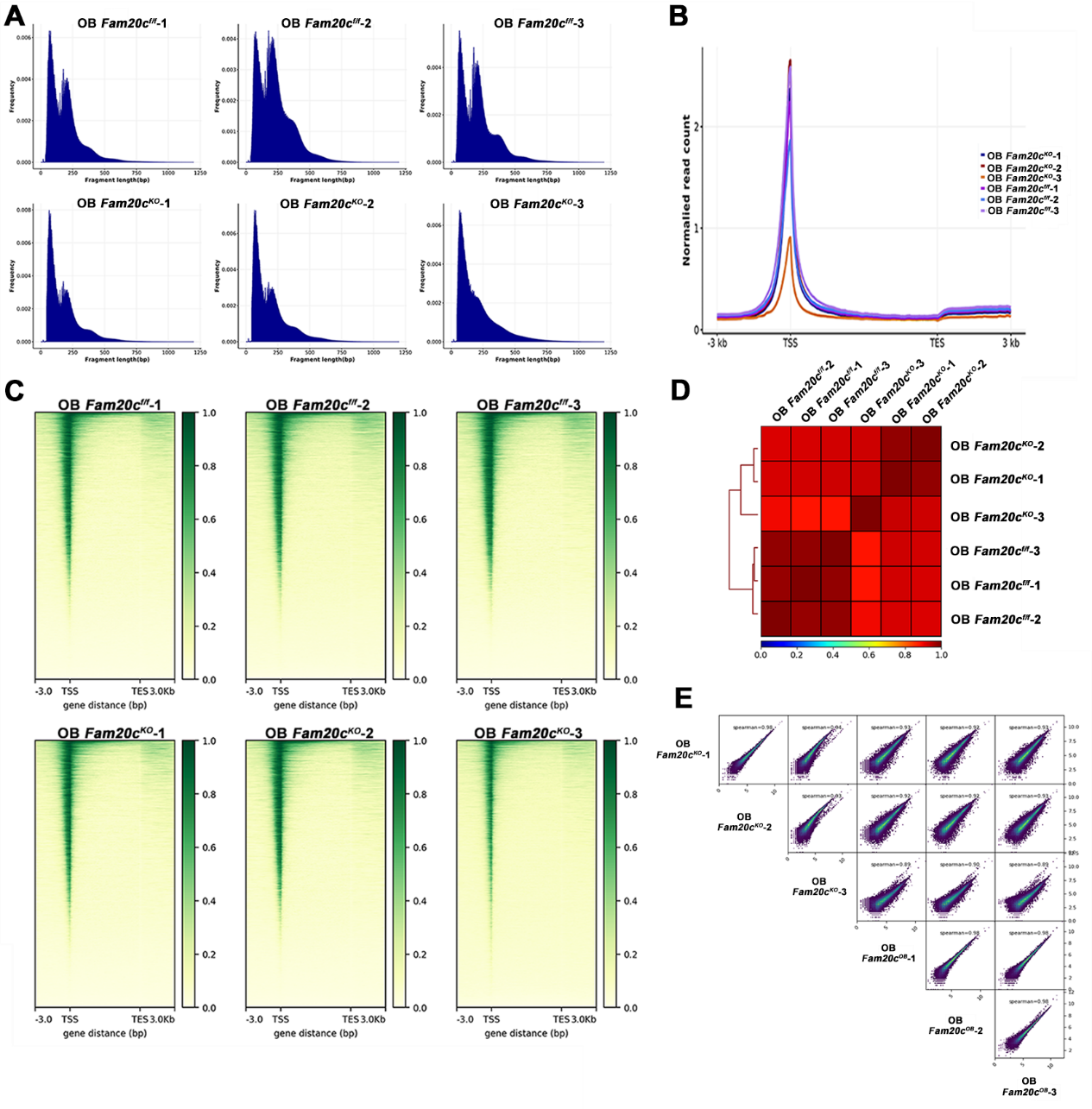


Figure S1 Quality control for ATAC-seq samples generated in this study.

A: Fragment length distribution map. The X-axis represents fragment length (bp), and the Y-axis represents frequency.

B: Reads distributions (from bigwig) across genes are presented as an average plot (average of reads signals across all genes).

C: Reads distributions (from bigwig) across genes are presented as a heatmap. The X-axis represents the normalized gene range coordinates, and the Y-axis represents the read enrichment. The larger the value, the more enriched. TSS stands for the gene start site, and TES stands for the gene stop site. -3.0 represents 3 kb upstream of TSS, and 3.0 kb represents 3 kb downstream of TES. Ensembl *Mus musculus* reference genome annotation (mm10_gencode) is used as regions for calculating enrichment of the ATAC signal at and around the TSS. The heatmap is made using deeptools.

D: The Pearson correlation results are shown by the heatmap.

E: The Pearson correlation results are shown by the heatmap scatterplot.
