# Supplementary material for: Fam20c regulates the calpain proteolysis system through phosphorylating Calpasatatin to maintain cell homeostasis
Source: J Transl Med. 2023 Jun 27;21:417. doi: 10.1186/s12967-023-04275-4 (PMC10294482; doi:10.1186/s12967-023-04275-4)
Supplement: Supplementary file 2 — Additional file 2. Fig. S2. Cell proliferation assay of OB Fam20cf/f and OB Fam20cKO vitro. [file 12967_2023_4275_MOESM2_ESM.docx]

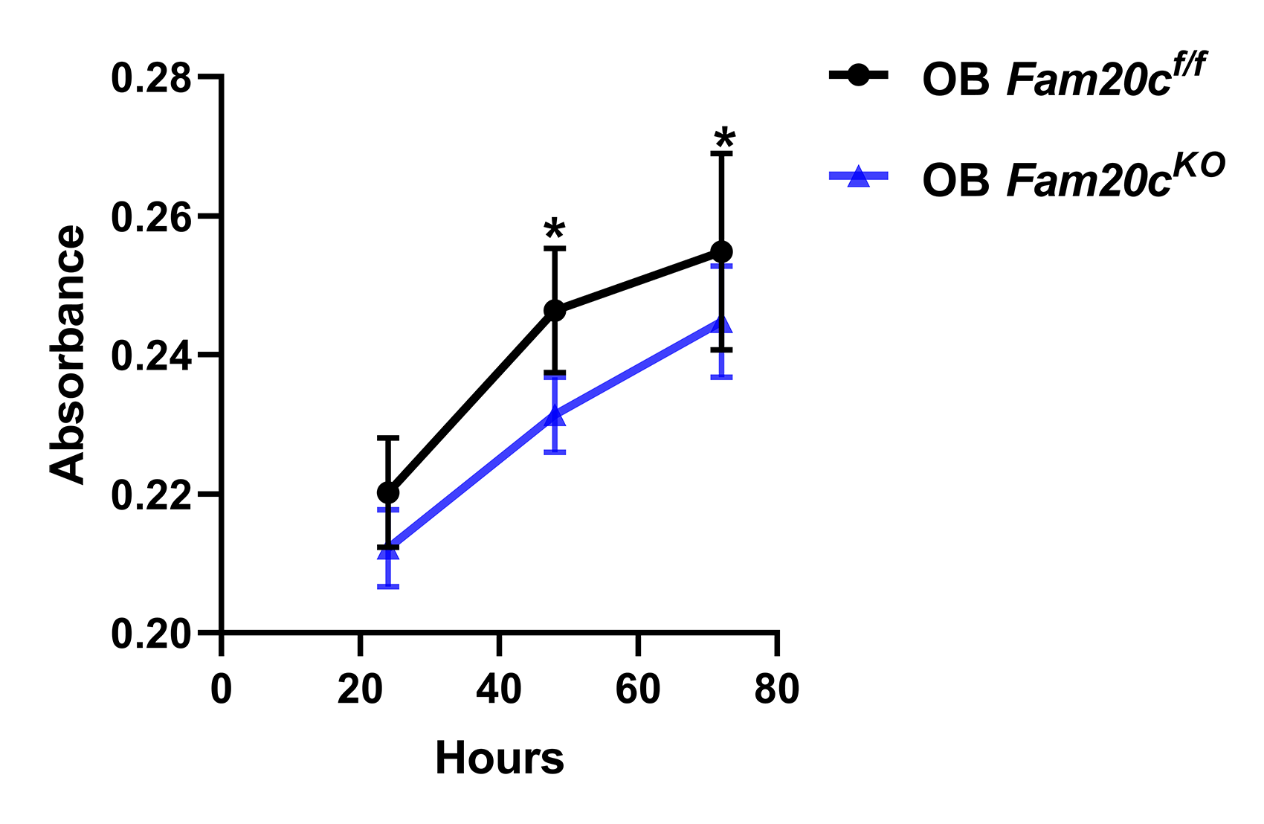


Figure S2 Cell proliferation assay of OB *Fam20c^f/f^* and OB *Fam20c^KO^* *vitro*.

X-axis represents hour, and the Y-axis represents absorbance. The black line represents OB *Fam20c^f/f^*, and the blue line represents OB *Fam20c^KO^*. **P* <0.05, *t* test.
