# Supplementary material for: Fam20c regulates the calpain proteolysis system through phosphorylating Calpasatatin to maintain cell homeostasis
Source: J Transl Med. 2023 Jun 27;21:417. doi: 10.1186/s12967-023-04275-4 (PMC10294482; doi:10.1186/s12967-023-04275-4)
Supplement: Supplementary file 3 — Additional file 3. Fig. S3 Cell proliferation assay and cytotoxicity assay by cell counting kit-8 in Fam20cKO treated with CaCl2. [file 12967_2023_4275_MOESM3_ESM.docx]

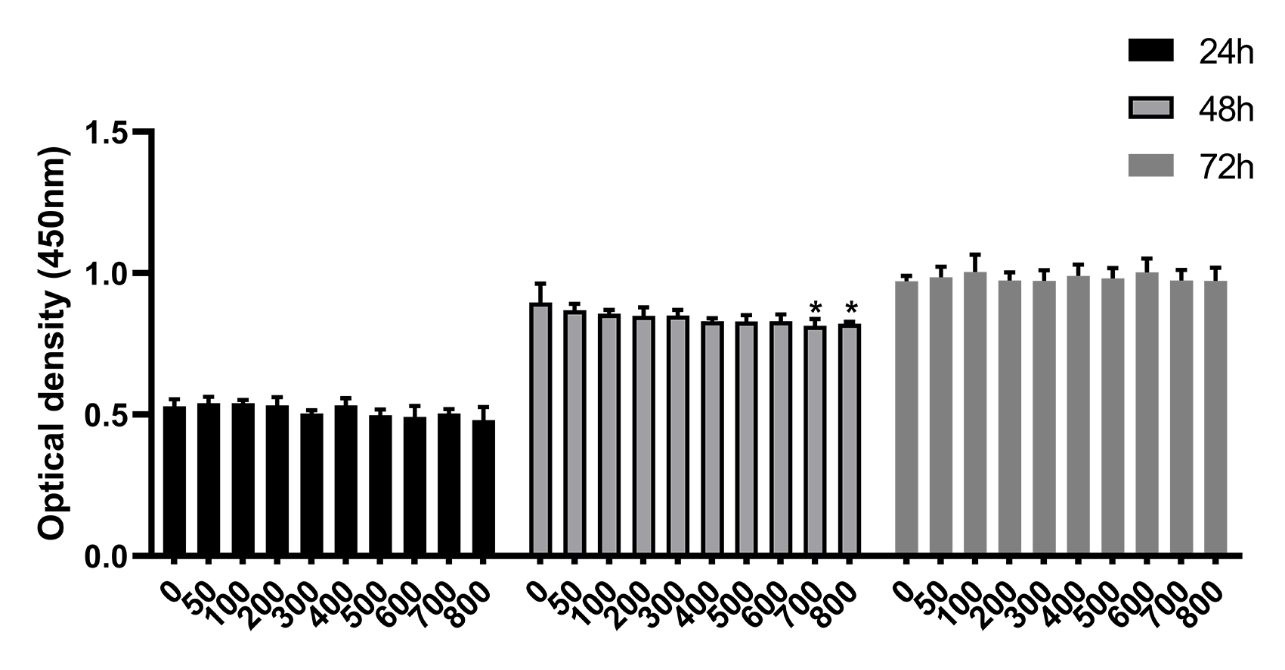


Figure S3 Cell proliferation assay and cytotoxicity assay by cell counting kit-8 in OB *Fam20c^f/f^* treated with CaCl_2_.

X-axis represents the concentration of CaCl_2_, and the Y-axis represents optical density. **P* <0.05, *t* test.
