# Supplementary material for: Fam20c regulates the calpain proteolysis system through phosphorylating Calpasatatin to maintain cell homeostasis
Source: J Transl Med. 2023 Jun 27;21:417. doi: 10.1186/s12967-023-04275-4 (PMC10294482; doi:10.1186/s12967-023-04275-4)
Supplement: Supplementary file 4 — Additional file 4. Fig. S4 Pathway map of overlapped genes from ATAC-seq and RNA-seq. [file 12967_2023_4275_MOESM4_ESM.docx]

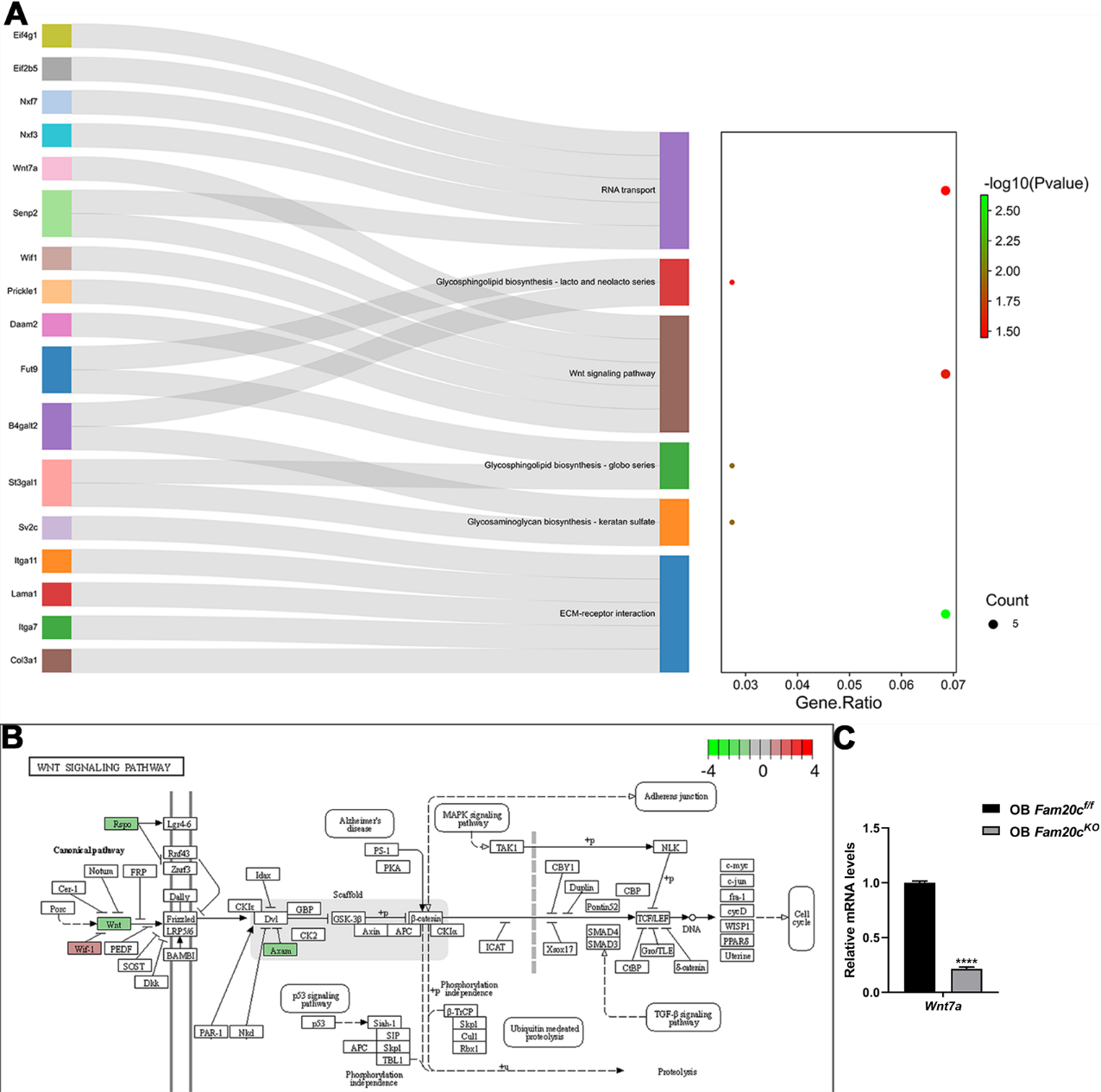


Figure S4 Pathway map of overlapped genes from ATAC-seq and RNA-seq.

A. Sankey diagram depicts the KEGG pathway within the intersection of genes with DAR-associated genes and DEGs in OB *Fam20c^KO^*.

B. Wnt pathway map depicts by the intersection of genes with DAR-associated genes and DEGs in OB *Fam20c^KO^*.

C. Expression (qPCR) of *Wnt7a* in OB *Fam20c^f/f^* and OB *Fam20c^KO^*. ^****^*P* < 0.0001.
