# Supplementary material for: Fam20c regulates the calpain proteolysis system through phosphorylating Calpasatatin to maintain cell homeostasis
Source: J Transl Med. 2023 Jun 27;21:417. doi: 10.1186/s12967-023-04275-4 (PMC10294482; doi:10.1186/s12967-023-04275-4)
Supplement: Supplementary file 5 — Additional file 5. Fig. S5 Kyoto Encyclopedia of Genes and Genomes (KEGG) pathway enrichment analysis based on ATAC-seq. [file 12967_2023_4275_MOESM5_ESM.docx]

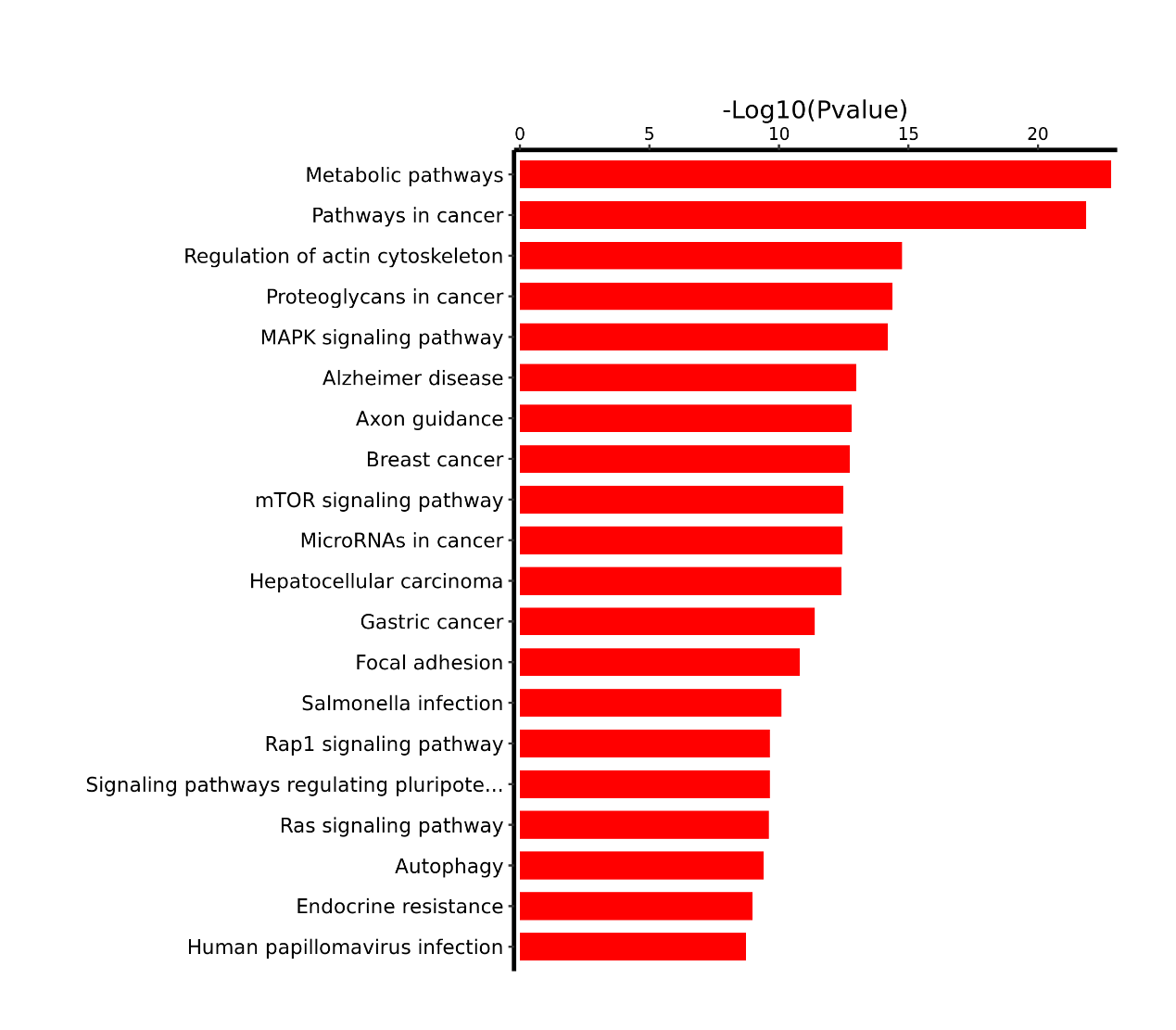


Figure S5 Kyoto Encyclopedia of Genes and Genomes (KEGG) pathway enrichment analysis based on ATAC-seq.

The horizontal axis indicates -log10(P value) and the vertical axis indicates the KEGG pathway name. The red color represents the significant items
