# Supplementary material for: Fam20c regulates the calpain proteolysis system through phosphorylating Calpasatatin to maintain cell homeostasis
Source: J Transl Med. 2023 Jun 27;21:417. doi: 10.1186/s12967-023-04275-4 (PMC10294482; doi:10.1186/s12967-023-04275-4)
Supplement: Supplementary file 6 — Additional file 6. Fig. S6 The changes of Calpastatin/Calpain proteolysis system in human 293T cells after Fam20c knock out [file 12967_2023_4275_MOESM6_ESM.docx]

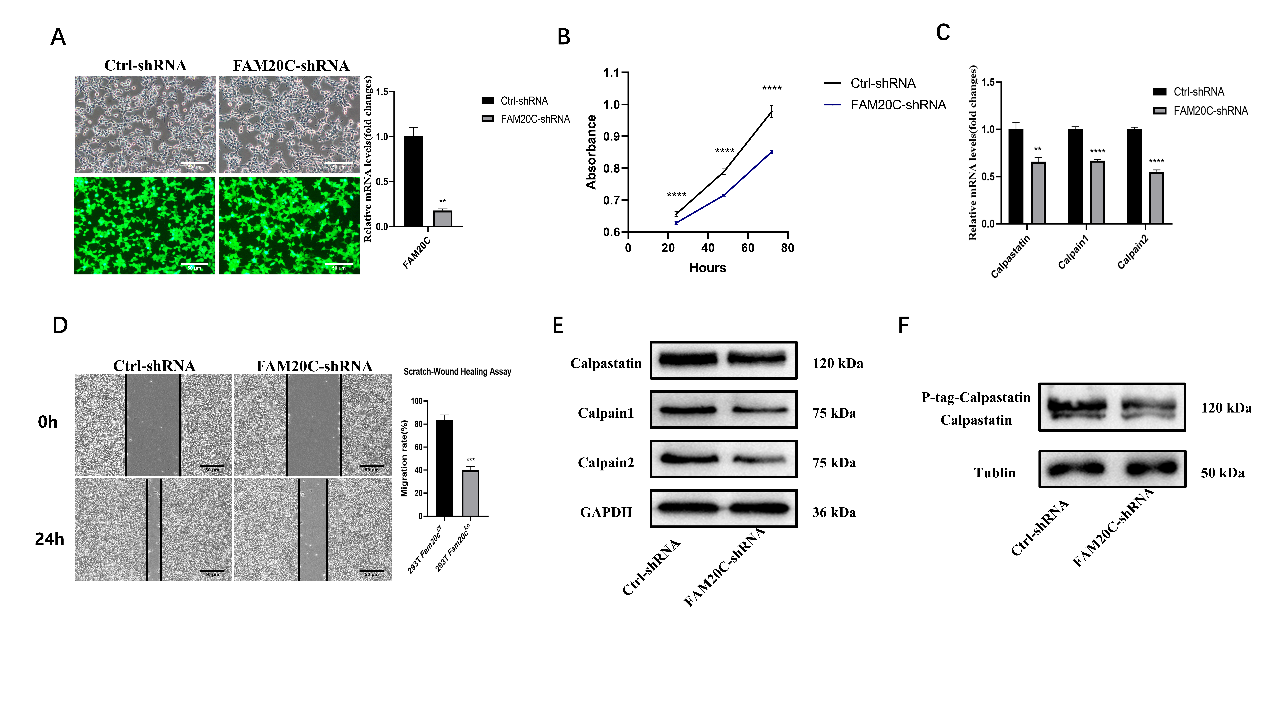


Figure S6 The changes of Calpastatin/Calpain proteolysis system in human 293T cells after *FAM20C* knock out.

A. Effect of lentivirus transfection FAM20C gene expression in human 293T cells.

B. Cell proliferation assay of Ctrl-shRNA and FAM20C-shRNA. ^****^*P* < 0.0001.

C. Gene expression of *Calpastatin*, *Calpain 1,* and *Calpain 2* in Ctrl-shRNA and FAM20C-shRNA. ^**^*P* < 0.01, ^****^*P* < 0.0001.

D. Wounding healing assays of Ctrl-shRNA and FAM20C-shRNA, images were acquired 0h and 24h after the scratch. ^***^*P* < 0.001.

E. Western blot analysis of Calpastatin, Calpain 1, and Calpain 2 expression in Ctrl-shRNA and FAM20C-shRNA. GAPDH served as an internal control.

F. Phos-Tag sodium dodecyl sulfate-polyacrylamide gel electrophoresis (SDS-PAGE) (P-tag) demonstrates Calpastatin phosphorylation levels in Ctrl-shRNA and FAM20C-shRNA. Tublin served as an internal control.
