# Supplementary material for: Fam20c regulates the calpain proteolysis system through phosphorylating Calpasatatin to maintain cell homeostasis
Source: J Transl Med. 2023 Jun 27;21:417. doi: 10.1186/s12967-023-04275-4 (PMC10294482; doi:10.1186/s12967-023-04275-4)
Supplement: Supplementary file 7 — Additional file 7. Table S1 ATAC-seq sequencing data quality control statistics. [file 12967_2023_4275_MOESM7_ESM.docx]

Table S1 ATAC-seq sequencing data quality control statistics.

| Sample  Name | Raw  Reads | Clean  Reads | Raw  Bases(G) | Clean  bases(G) | Q20  (%) | Q30  (%) | GC Content  (%) |
| --- | --- | --- | --- | --- | --- | --- | --- |
| OB *Fam20c^f/f^*-1 | 86973918 | 85439586 | 13.05 | 10.39 | 98.05 | 93.88 | 49.61 |
| OB *Fam20c^f/f^*-2 | 133000000 | 130864992 | 19.95 | 17.07 | 98.41 | 94.62 | 47.74 |
| OB *Fam20c^f/f^*-3 | 159277568 | 156631758 | 23.89 | 19.60 | 98.31 | 94.57 | 50.55 |
| OB *Fam20c^KO^*-1 | 142811506 | 138743096 | 21.42 | 15.89 | 98.19 | 94.05 | 51.02 |
| OB *Fam20c^KO^*-2 | 175112460 | 170766974 | 26.27 | 20.07 | 98.11 | 93.67 | 50.91 |
| OB *Fam20c^KO^*-3 | 108901140 | 106424542 | 16.34 | 12.59 | 97.95 | 93.07 | 45.25 |
