# Supplementary material for: Fam20c regulates the calpain proteolysis system through phosphorylating Calpasatatin to maintain cell homeostasis
Source: J Transl Med. 2023 Jun 27;21:417. doi: 10.1186/s12967-023-04275-4 (PMC10294482; doi:10.1186/s12967-023-04275-4)
Supplement: Supplementary file 8 — Additional file 8. Table S2 Statistics of the alignment results of Reads on the reference genome sequence. [file 12967_2023_4275_MOESM8_ESM.docx]

Table S2 Statistics of the alignment results of Reads on the reference genome sequence.

| Statistics | All | UnMapped | Mapped | Mapped Rate | Unique  Mapped | Unique Mapped Rate | MultiMapped |
| --- | --- | --- | --- | --- | --- | --- | --- |
| OB *Fam20c^f/f^*-1 | 85613308 | 165889 | 85447419 | 0.998 | 79892877 | 0.933 | 5554542 |
| OB *Fam20c^f/f^*-2 | 131179680 | 206712 | 130972968 | 0.998 | 121672786 | 0.928 | 9300182 |
| OB *Fam20c^f/f^*-3 | 156972060 | 353390 | 156618670 | 0.998 | 147996816 | 0.943 | 8621854 |
| OB *Fam20c^KO^*-1 | 139044470 | 280528 | 138763942 | 0.998 | 128833616 | 0.927 | 9930326 |
| OB *Fam20c^KO^*-2 | 171135022 | 384854 | 170750168 | 0.998 | 158433157 | 0.926 | 12317011 |
| OB *Fam20c^KO^*-3 | 106671581 | 130273 | 106541308 | 0.999 | 94712010 | 0.888 | 11829298 |
